# Infection of domestic pigs with a genotype II potent strain of ASFV causes cytokine storm and lymphocyte mass reduction

**DOI:** 10.3389/fimmu.2024.1361531

**Published:** 2024-04-18

**Authors:** Xuezhi Zuo, Guorui Peng, Junjie Zhao, Qizu Zhao, Yuanyuan Zhu, Yuan Xu, Lu Xu, Fangtao Li, Yingju Xia, Yebing Liu, Cheng Wang, Zhen Wang, Haidong Wang, Xingqi Zou

**Affiliations:** ^1^ China/WOAH Reference Laboratory for Classical Swine Fever, China Institute of Veterinary Drug Control, Beijing, China; ^2^ College of Veterinary Medicine, Shanxi Agricultural University, Jinzhong, Shanxi, China

**Keywords:** ASFV, inflammatory, pigs, immune, vaccine

## Abstract

The whole-genome sequence of an African swine fever virus (ASFV) strain (HuB/HH/2019) isolated from Hubei, China, was highly similar to that of the Georgia 2007/1 strain ASFV. After infection with strong strains, domestic pigs show typical symptoms of infection, including fever, depression, reddening of the skin, hemorrhagic swelling of various tissues, and dysfunction. The earliest detoxification occurred in pharyngeal swabs at 4 days post-infection. The viral load in the blood was extremely high, and ASFV was detected in multiple tissues, with the highest viral loads in the spleen and lungs. An imbalance between pro- and anti-inflammatory factors in the serum leads to an excessive inflammatory response in the body. Immune factor expression is suppressed without effectively eliciting an immune defense. Antibodies against p30 were not detected in acutely dead domestic pigs. Sequencing of the peripheral blood mononuclear cell transcriptome revealed elevated transcription of genes associated with immunity, defense, and stress. The massive reduction in lymphocyte counts in the blood collapses the body’s immune system. An excessive inflammatory response with a massive reduction in the lymphocyte count may be an important cause of mortality in domestic pigs. These two reasons have inspired researchers to reduce excessive inflammatory responses and stimulate effective immune responses for future vaccine development.

## 1 Introduction

African swine fever virus (ASFV) has a large and complex genome. It is the only member of the genus ASFV in the family ASFV and is the only known arbovirus (1). Its genome size is 170–190 kb, including 151–167 open reading frames (ORFs) (2). It can be classified into 24 genotypes based on the B646L gene (3, 4).

ASFV predominantly infects the monocyte-macrophage system, including circulating monocytes, intra-tissue macrophages, and dendritic cells, providing a favorable environment for ASFV replication and infection. These cells play an important role in host innate and adaptive immunity; therefore, ASFV can inhibit host immunity and evade innate and adaptive immune responses (5). It can evade host immunity through multiple pathways, which mainly include the inhibition of IFN secretion through signaling pathways such as cGAS-STING and JAK-STAT (6–10); moreover, it inhibits IFN secretion through signaling pathways such as NF-κB to impede the host inflammatory response (11, 12) and the apoptosis of infected cells (13–15).

ASFV-infected hosts lacking certain genes can develop immune protection and defend against their original strains, such as ASFV/CN/GS/2018-Δ9L/Δ7R, ASFV-G-ΔA137R, and ASFV-G-ΔA151R, although the virus can still be detected in the blood (16–18). The absence of these genes provides effective protection; however, their safety is a major concern. Neutralizing antibodies produced by ASFV-stimulated hosts are effective against weak strains but are insufficient to produce robust protection against strong strains (19). It is important to understand the effects of the original strain on the host immune system to develop effective vaccines.

This study used HuB/HH/2019 genotype II ASFV-infected domestic pigs and collected pharyngeal swabs, anal swabs, tissues, blood, serum, and peripheral blood mononuclear cells (PBMCs) to analyze the dynamic changes in carriage and detoxification, blood viral load, cytokines, PBMCs transcripts, and immune cells in domestic pigs with the infection.

## 2 Materials and methods

### 2.1 Viral virulence determination and whole-genome sequencing

Each dilution of HuB/HH/2019 ASFV and the negative and positive controls were sequentially added to PAM 96-well cell plates, and 20 μL of fresh 1% porcine erythrocytes were added to each well and incubated in a 37°C, 5% CO_2_ incubator. The plates were observed every day to see whether the erythrocyte adsorption phenomenon (HAD) occurred until the positive control showed HAD. The results were calculated as HAD_50_ using the method proposed by Reed and Muench (20).

Nucleic acids were extracted from HuB/HH/2019 strain ASFV according to the instructions of the QIAamp DNA Mini Kit (Qiagen, Germany). 200µl of the sample was mixed with 20µl of QIAGEN protease from the kit and 200µl of buffer AL with sufficient shaking and then incubated for 10 min at 56°C to allow for sufficient cleavage of the sample. Subsequently, 200µl of anhydrous ethanol was added, mixed thoroughly and then added to the QIAamp Mini rotary column (into a 2ml collection tube) and centrifuged at 6000g (8000rpm) for 1 min and the liquid was discarded. Then, 500μl Buffer AW1 was added and it was centrifuged at 6000g (8000rpm) for 1 min and the liquid was discarded liquid. We then added500μl of Buffer AW2 and centrifuged at 20,000g (14,000rpm) for 3 min, discarding the liquid and the collection tube. The QIAamp Mini Spin Column was placed into a new 2 ml collection tube and centrifuged at full speed for 1 min to help eliminate residual Buffer AW2. The QIAamp Mini Spin Column was placed into a new 1.5 ml centrifuge tube with 50 μl of Buffer AE, incubated for 1 min at room temperature (15-25°C), and centrifuged at 6,000g (8,000 rpm) for 1 min to elute the DNA. The DNA was eluted by centrifugation at 6000 g (8000 rpm) for 1 min. To improve the recovery rate, the eluted DNA was added to the QIAamp Mini rotary column again and centrifuged by incubation at room temperature. The extracted nucleic acids were subjected to whole genome sequencing.

The strain used in the present experiment was isolated during an outbreak in Hubei Province, China, in July 2019. The strain has been reported in the Veterinary Bulletin of the Ministry of Agriculture and Rural Affairs of China, Volume 21, Issue 8, 2019, in addition to the World Organization for Animal Health, Reference Laboratory for Classical Swine Fever, China Institute of Veterinary Drug Control, Beijing, China.

### 2.2 Animal experiments

All animal experiments were approved by the Animal Welfare and Ethics Committee of the China Veterinary Drug Inspection Institute (IVDC), and animal experiments and virus sampling were conducted in a biosafety level III laboratory of the IVDC, approved by the Ministry of Agriculture and Rural Affairs of the People’s Republic of China.

Nine 60-day-old Dugongs (ternary pigs) were divided into two groups: six in the inoculation group and three in the negative control group. Before inoculation, their blood was tested for ASFV, PEDV, TGEV, CSFV, PPV, PCV II, PRV, and PRRSV antigens. All results were negative. The inoculation group was intramuscularly injected with the 10HAD_50_ HuB/HH/2019 strain of ASFV in the neck (no. 2#, 3#, 5#, 7#, 8#, and 9#).

Their temperature was recorded daily to observe clinical signs and scored, expressed as a quantitative clinical score (CS) obtained by summing the values of the eight clinical signs recorded daily, namely, temperature, anorexia, flatus, skin hemorrhage or cyanosis, joint swelling, respiratory distress, ocular discharge, and the digestive system. The findings were scored on a severity scale of 0–3 (most severe), and the sum of these points was recorded as CS, which was also used to define human endpoints (21). Pigs with a clinical score greater than 15 were culled under anesthesia using 1 ml of Zoletil 50, indicating that five or more of the eight clinical signs recorded reached the most severe stage and that three pigs with a score greater than 15 died naturally in the subsequent experiment. The period after the onset of typical signs and natural death or culling was defined as the near-death period, during which the disease progressively deteriorated less than 3 days before natural death and culling. Blood and swabs were collected 0, 2, 4, 6, and 8 days post-inoculation (dpi) in the inoculated group. PBMCs were isolated for transcriptome sequencing and analysis of changes in immune cell subsets. Their serum was isolated for cytokine expression assays. The pigs that survived were observed until day 21 of the necropsy, and tissues were collected for ASFV nucleic acid testing.

### 2.3 Carrying and detoxification pattern after infection with HuB/HH/2019 strain of ASFV

Blood was diluted 10-fold with PBS before nucleic acid extraction, with pharyngeal swabs and anal swabs centrifuged to obtain supernatants. Small tissue samples were taken (soybean-sized) and ground with 1 mL of PBS, and the resulting mixture was centrifuged to obtain supernatants. All samples were processed, and nucleic acids were extracted using the Tecan nucleic acid workstation and magnetic bead method viral DNA/RNA extraction kit (Beijing, China). The primer probe sequences are shown in **Table 1** (22). A 25-μl system was prepared, including HyperProbe Mixture 12.5 μl, upstream primer (20 μM) 0.2 μl, downstream primer (20 μM) 0.2 μl, probe (10 μM) 0.1 μl, nucleic acid 3 μl, and ddH2O 9 μl. The amplification program was pre-denaturation at 95°C for 30s, denaturation at 95°for C 10s, annealing/extension at 58°C for 20s for 45 cycles.

**Table 1:** p72 primer/probe sequences.

p72	Sequences
Upstream Primer	5’-GAACGTGAACCTTGCTA-3’
Downstream Primer	5’-GGAAATTCATTCACCAAATCC-3’
Probe	5’-6-FAM-TAAAGCTTGCATCGCA-MGB-3’

### 2.4 Dynamic changes in serum antibodies with different days of infection

Changing levels of p30 antibody in serum with days of infection were measured using an indirect ELISA (JNT, China), and all components were removed from the kit and returned to room temperature. To the wells of the antigen-coated plate, 100 μl of extracted samples diluted in a dilution plate were added (samples were diluted 50-fold using sample diluent). Next, 100 μl of undiluted negative control serum (NC) and positive control serum (PC) were added to the indicated wells. The plate sealing membrane was covered and incubated for 30 min ( ± 2 min) at room temperature (25 ± 3°C). The plate was washed with a micropipette, the liquid in the wells was discarded, and 300 μl of 1x washing solution was added to each well, and washed three times. After the last washing, the plate was gently patted dry on absorbent paper, and the drying of the plate between steps was strictly prohibited. Next, 100μl ASFV HPR labeled antibody was added to each well. The plate sealing membrane was covered and incubated for 30min ( ± 2min) at room temperature (25 ± 3°C). The plate was washed with a micropipette, the liquid was discarded in the wells, and 300 μl of 1x washing solution was added to each well, and washed three times. After the last washing, the plate was gently patted dry on absorbent paper, and drying of the plate between steps was strictly prohibited. At this point, 100μl TMB substrate solution was added to each well. The plate was covered with a sealing membrane and incubated for 15min ( ± 1min) at room temperature (25 ± 3°C). The enzymatic reaction was terminated by adding 50 μl of termination solution to each well. Absorbance values were measured using a wavelength of 450 nm. Results were determined and calculated.

The conditions for the establishment of the experiment were positive control OD450 > 0.8 and negative control OD450 < 0.2. The formula for calculating the S/P of the samples was (OD value of the samples - mean value of the OD of the negative control)/(mean value of the OD of the positive control - mean value of the OD of the negative control). The criteria for determining the S/P of positive samples were positive S/P ≥ 0.4, suspicious 0.4 > S/P > 0.3, and negative S/P ≤ 0.3, and the suspicious samples were tested again to confirm these results.

### 2.5 Changes in serum cytokine dynamics under different days of infection

Serum was collected and assayed using Luminex for changes in the concentrations of IFN-α, IFN-γ, IL-1β, IL-4, IL-6, IL-8, IL-10, IL-12p40, and TNF-α with different days of infection. The standards were dissolved and the components diluted according to the instructions of the kit (Thermo, USA, EPX090-60829-901). Next, 50 μl of pre-mixed microspheres was added to a 96-well plate and the plate was washed with a magnetic separator. The 96-well plate was taken out and 25 μl of Universal Assay Buffer was added to each well. Next, 25 μl of standards or samples was added; 25 μl of Universal Assay Buffer was then added to the blank control; and the plate was sealed with a membrane and incubated for 30 min at 500 rpm at room temperature with shaking. Afterward, 25μl of Universal Assay Buffer was added to the blank control; the membrane of the well plate was sealed, incubated for 30min at 500rpm with shaking at room temperature, and left at 4°C overnight. The plate was removed the next day and incubated with shaking at 500 rpm for 30 min at room temperature. The 96-well plate was placed in a magnetic separator plate, 150 μl of 1×wash buffer was added to each well, and it was allowed to stand for 30 s. The liquid from the well plate was removed by inverting it. The steps were repeated for a total of 3 washes; at the end of the last wash, the residual liquid was adsorbed with a paper towel. Next, 25 μl of 1× detection antibody mixture was added to each well; the plate was sealed with a new sealing film; the 96-well plate was removed from the magnetic separator plate and placed in a plate shaker at 500 rpm for 30 min at room temperature; the washing procedure was repeated. Then, 50μl of SA-PE was added to each well; the plate was sealed with a new sealing membrane; the 96-well plate was removed from the magnetic separator plate and placed in a well plate shaker at 500rpm for 30min at room temperature. the plate-washing step was repeated. Next, 120 μl of Reading Buffer was added to each well; the plate was sealed with a new sealing membrane; the 96-well plate was removed from the magnetic separator plate and placed in a well plate shaker at 500 rpm for 5 min; the sealing membrane was gently removed and placed in a Luminex 200 instrument for reading. The standard curve was fitted by five-parameter nonlinear regression and the concentration values were calculated.

### 2.6 RNA-sequencing and quantitative polymerase chain reaction analysis of PBMCs transcriptional differences in different days of infection

PBMCs were isolated using Porcine Peripheral Blood Single Nucleated Cell Isolation Kit (IPHASE, China). 10 ml of blood was taken from the jugular vein and added to the EDTA anticoagulation tube, 10 ml of 1× dilution solution pre-diluted with ultrapure water was added to a 50 ml centrifuge tube and 10 ml of blood was added. The 20 ml of diluted blood was spread into a 50 ml centrifuge tube containing 10 ml of separating solution, while ensuring that the liquid level was stratified. After centrifugation at 800 g for 20 min, a flocculent white membrane layer was visible. The white membrane layer was aspirated with a pasteurized pipette and added to the centrifuge tube containing 10 ml of diluent solution by centrifugation at 400 g for 10 min and the supernatant was discarded. The white membrane layer was removed from the vein and added to the EDTA anticoagulant tube. The precipitate was suspended with 10 ml of erythrocyte lysate and then centrifuged at 400 g for 10 min. The precipitate was suspended in cryopreservation solution, part of which was frozen and the other part was added to RNAiso Plus (Invitrogen, USA) for RNA-seq sequencing.

A total of five groups were set up for RNA-seq sequencing, including the negative control (0 dpi), P0521 (2 dpi), P0523 (4 dpi), P0525 (6 dpi), and P0527 (8 dpi) groups. The Illumina II high-throughput sequencing platform was utilized with the PE150 sequencing strategy. Comparison and transcript splicing analyses were completed using star and Cufflinks software, respectively, and then all genes were quantitatively analyzed to identify the differential genes, which were subsequently subjected to functional enrichment analyses to mine their functions.

Based on the RNA-seq results, six chemokines (**Table 2**) were selected for qPCR validation. PMBCs with RNAiso Plus were thawed, chloroform was added and shaken gently, and allowed to stand on ice for 7 min, and delamination could be observed. Centrifugation was performed at 12,000 rpm for 15 min, and the supernatant was aspirated into a new 1.5 mL centrifuge tube, isopropanol was added and shaken gently, and allowed to stand on ice for 15 min. Centrifugation was performed at 12,000 rpm for 15 min, and the supernatant was poured off. The precipitate was washed by adding pre-cooled 75% ethanol (anhydrous ethanol configured with depc water) at 4°C. It was then centrifuged at 8000rpm for 8min, after which the supernatant was poured off, and the wells left to air-dry to add depc water to solubilize the RNA.

**Table 2:** Sequences of chemokine primers.

Chemokine Name	Forward (5,)	Reverse (3,)
CXCL14	ACCCAGCACTTTTACCGAGG	TGTCTGGAGCGCAAGAGAAG
CXCL11	AATACCACTGCCCAGAGTAGC	ATACCCAGTTGGGAACCAGC
CXCL9	CTCAGCTTTTCCCGCAGAGT	TTGGTGGCCTTCTTGTCAGG
CXCL10	ACTGATAAGGATGGGCCGGA	TTACTGCTCAACAGCTCGGG
CCL2	GATCTTCAAGACCATCGCGG	GGTTTTTCTTGTCCAGGTGGC
CCL21	GCTATGTGCAGACCCCCAAA	ATTTGGAGGCCCTCTTGTCC

The extracted RNA was tested for purity and quality using a NanoDrop 1000 (Thermo, USA), and qualified RNA was mixed with PrimeScript™ RT Master Mix (Takara,Japan) at a ratio of 1:4, and reverse transcribed into cDNA at 37°C for 15 min and 85°C for 5 s. The cDNAs were extracted from the RNAs using a NanoDrop 1000 (Thermo, USA).

The internal reference gene for qPCR validation was β-actin. 10 μl of MonAmp™ SYBR^®^ Green qPCR Mix (Monad, China), 0.4 μl each of F/R, 2 μl of cDNA, 7.2 μl of ddH2O, and 20 μl of the total system were used to set up the reaction system. In the LightCycler 480II fluorescent qPCR instrument (Roche Holding AG, Switzerland), the program was set to pre-denaturation at 95°C for 30s, amplification at 95°C for 10s, 57°C for 15s, 72°C for 15s for a total of 45 cycles, and the lysis curve at 95°C for 10s, 65°C for 1min, 95°C for Continuous, and 40°C for None. The results were analyzed using the 2^-ΔΔCt^ method.

### 2.7 Immune cell dynamics in PBMCs at different days of infection

The collected PBMCs were centrifuged at 350 g for 5 min, and the supernatant was discarded. The precipitate was suspended in 300 μL FBS Buffer (BD, USA) and centrifuged; this step was repeated once. Next, 4% paraformaldehyde was fixed and washed 3 times. Subsequently, cells were suspended with 100 μl FBS Buffer containing 2 μl CD3-FITC (Abcam, UK,ab34722), 10 μl CD4-Percp/cy5.5 (BD, USA,561474), and 10 μl CD8-PE (Abcam, UK,ab22548). Next, the cells were incubated at room temperature and protected from the light for 15 min, washed twice, and then, suspended with 100 µL FBS buffer for on-board testing. Flow cytometry was performed using the CytoFLEX SRT system (Beckman Coulter, USA).

### 2.8 Statistical analyses

All data are presented as individual or mean+SEM, plotted using Prism 9 (GraphPad, San Diego, CA, USA), with statistically significant differences determined using one-way ANOVA and Bonferroni’s correction. P < 0.05 (*), P < 0.01 (**), P < 0.001 (***), and P < 0.0001 (****).

## 3 Results

### 3.1 Whole-genome sequence of the HuB/HH/2019 strain

**Figure 1:**
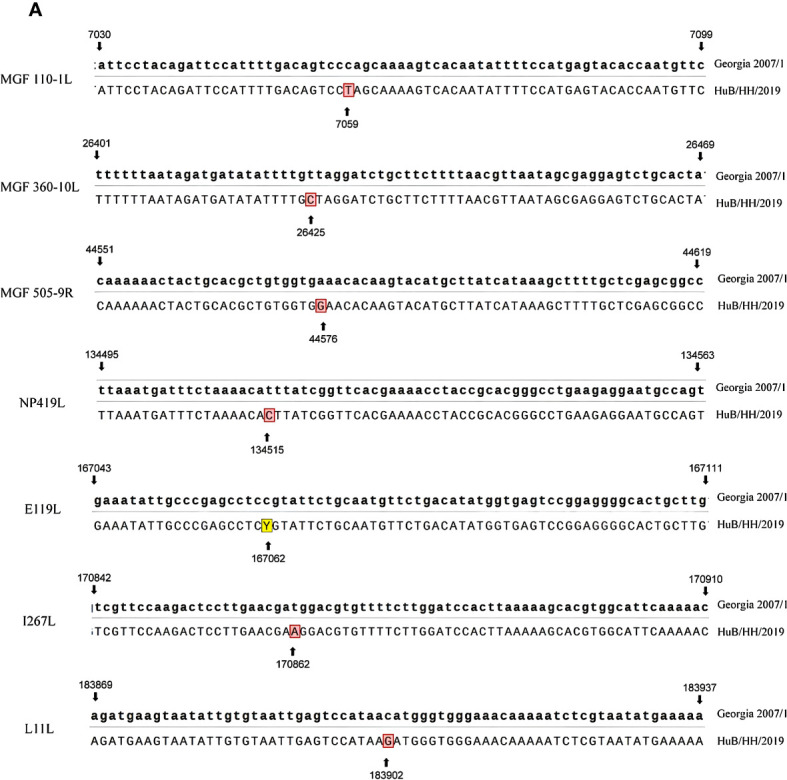
Sequence comparison of Georgia 2007/1 with HuB/HH/2019, with Georgia 2007/1 at the top and HuB/HH/2019 at the bottom.

Whole-genome sequence comparison between the HuB/HH/2019 strain ASFV and Georgia 2007/1 strain ASFV (NCBI GenBank: FR682468.2) revealed that the two strains were highly homologous, with seven base mismatches. The positions of the mismatches were 7059, 26425, 44576, 134514, 167062, 170862, and 183902 in Georgia 2007/1 ASFV (**Figure 1**).

### 3.2 Clinical scoring and necropsy observations

**Figure 2:**
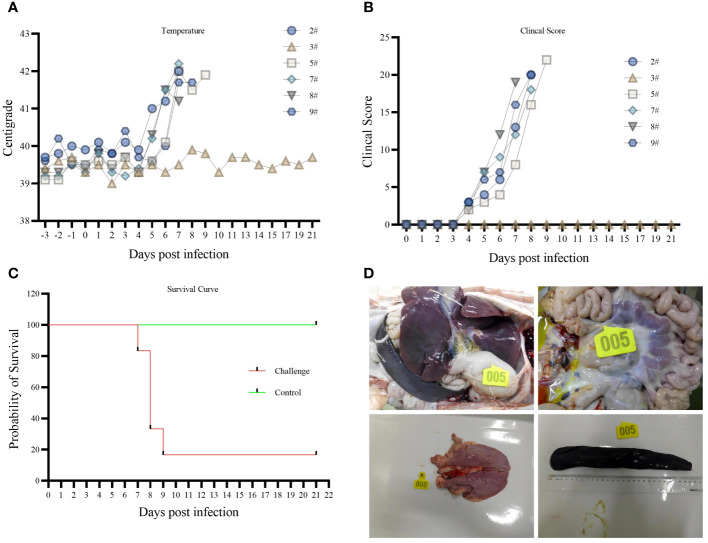
**(A)** body temperature variation. **(B)** Clinical Score. **(C)** survival curve. **(D)** Histopathology.

The body temperatures of the five pigs started to rise at 5 dpi, and all reached above 40°C at 6 dpi (**Figure 2A**). Three pigs died naturally at 8 dpi. Two had typical symptoms at 8 and 9 dpi and were dissected when they entered the near-death stage. Pig 3# remained alive till 21 dpi without elevated body temperature and obvious clinical symptoms (**Figures 2B, C**). All morbid pigs showed signs of generalized redness, huddling, decreased appetite, depression, recumbency, and shortness of breath (**Figures 2B, C**). The control group did not show a significant increase in body temperature, and no abnormal clinical signs were observed.

An autopsy of the affected pigs revealed inflamed submandibular, gastrohepatic, inguinal, and pulmonary hilar lymph nodes, hemorrhagic swelling of the tonsils, solid changes in the lungs, hemorrhages in the intestinal tract, and obvious enlargement of the spleen. Among them, five pigs had jelly-like liver sludge in their liver capsules (**Figure 2D**). Pig 3# did not develop any disease during the 21-day observation period. Slight hemorrhagic swelling of the spleen and lungs was observed through autopsy.

### 3.3 The pattern of virus excretion and virus tissue distribution

**Figure 3:**
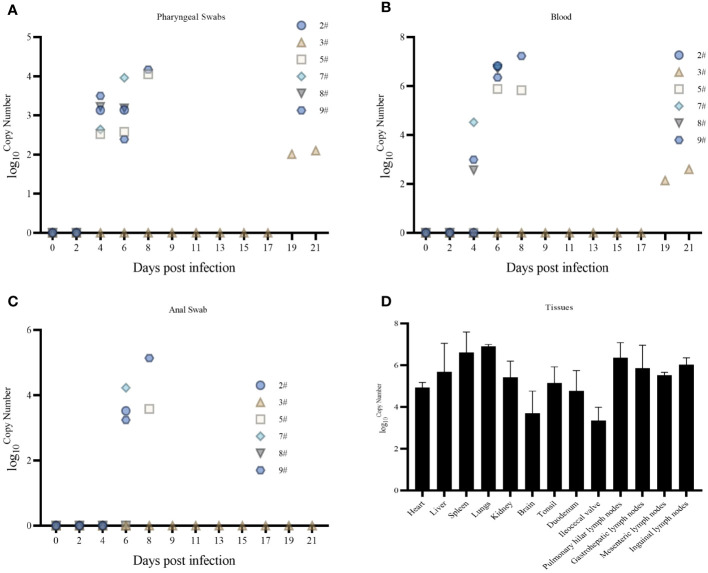
**(A)** Pharyngeal swab viral nucleic acid load. **(B)** Blood Viral Nucleic Acid Volume. **(C)** Anal swab viral nucleic acid load. **(D)** Tissue viral nucleic acid load.

Pharyngeal swabs, anal swabs, and blood were collected on different days after infection to detect the pattern of virus exocytosis and viremia over time, and the tissues were collected by dissection to detect the distribution of the virus; viral nucleic acids began to be detected in pharyngeal swabs at 4 dpi (**Figure 3A**), blood at 4 dpi (**Figure 3B**), and anal swabs at 6 dpi (**Figure 3C**). The amount of detoxification carryover gradually increased with the number of days of infection, and the amount of nucleic acids in the blood was higher than that in the swabs collected on the same day. Viral nucleic acids were detected in all 13 tissues collected (heart, liver, spleen, lungs, kidneys, brain, tonsils, duodenum, ileocecal valve, hilar lymph nodes, gastrohepatic lymph nodes, mesenteric lymph nodes, and inguinal lymph nodes) (**Figure 3D**). The lungs and spleen had the highest viral loads, and the ileocecal valve had the lowest viral load. Nucleic acids were only detected at very low levels in the spleen and lungs of Pig #3 (results not shown).

### 3.4 Serum antibody levels

**Figure 4:**
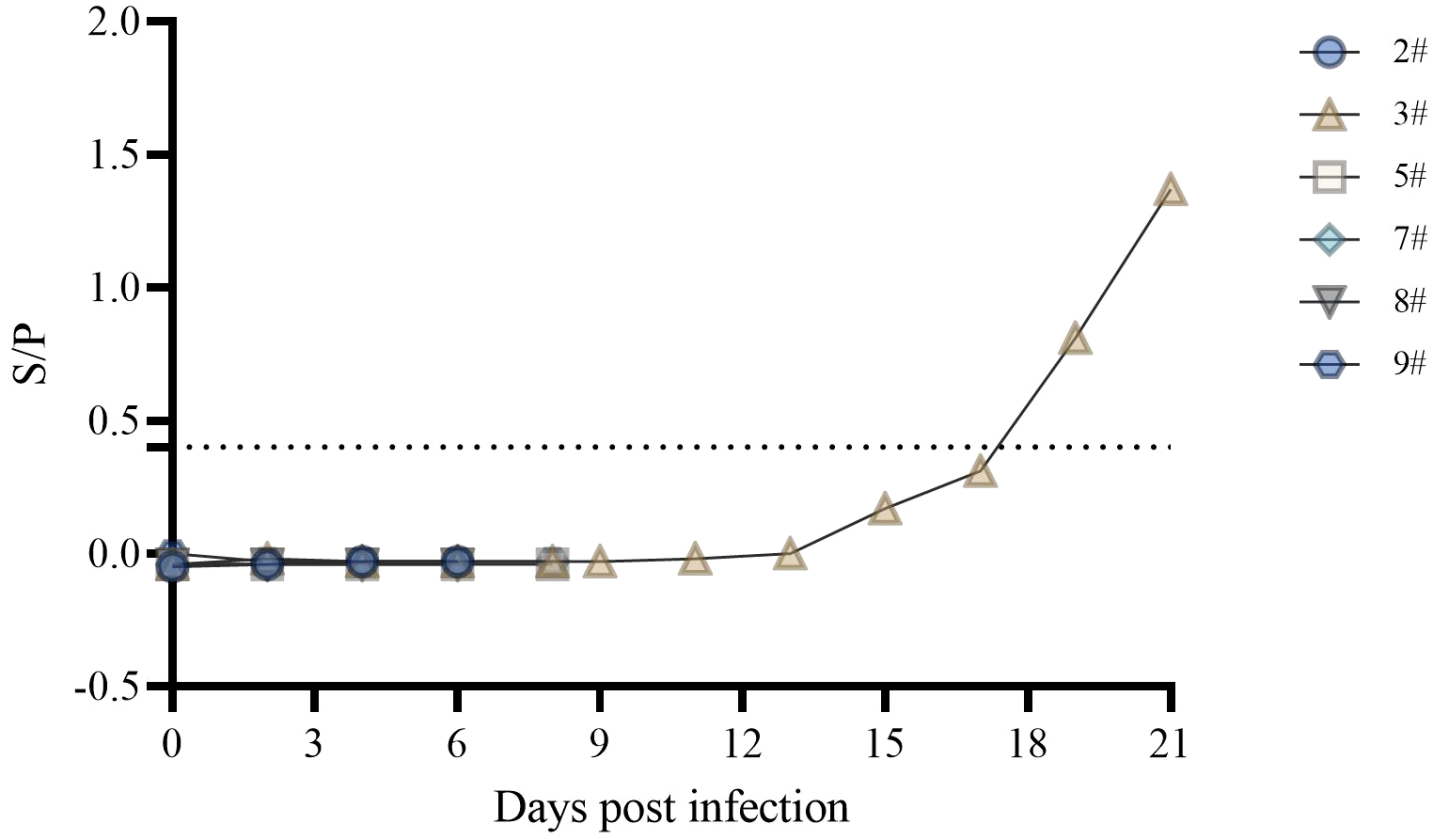
Expression of serum p30 antibody.

The ASFV p30 protein is an early expressing protein, detected in one of the six swine serum samples starting at 19 dpi (**Figure 4**). Although pig #3 did not show symptoms throughout the observation period, the presence of antibodies in the body indicated a low level of infection. In contrast, nucleic acid testing of swabs, blood, and tissues showed a lower level of carryover detoxification than in the rest of the pigs. This indicates the presence of infection in #3, but it was in good condition throughout the observation period. In a related study, p72 antibodies were detected in the serum of only one pig that survived for 12 days after infection of 16 domestic pigs with the Georgia 2007/1 strain of ASFV, whereas p72 antibodies were not detected in the remaining pigs that survived for 9 days (23).

### 3.5 Cytokine storm caused by ASFV infection

To better demonstrate the cytokine changes in domestic pigs, we categorized and analyzed them according to different life processes.

**Figure 5:**
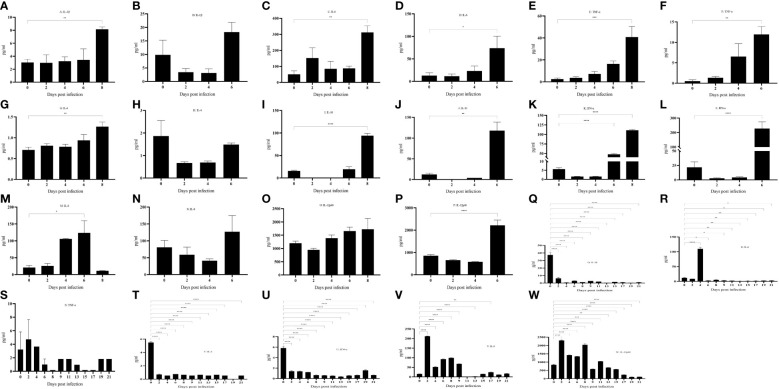
**(A–W)** Cytokine changes in different life processes. P < 0.05 (*), P < 0.01 (**), P < 0.001 (***), and P < 0.0001 (****).

No significant changes were found in the expression of pro-inflammatory factors IL-1β (**Figures 5A, B**) and IL-6 (**Figures 5C, D**) during the incubation period, but the expression increased significantly during the near-death period. The expression of pro-inflammatory factor TNF-α (**Figures 5E, F**) gradually increased after the attack. The expression of the anti-inflammatory factor IL-4 (**Figures 5G, H**) was not significantly different in domestic pigs with a life cycle of 6 days. In contrast, it was significantly elevated during the near-death period in domestic pigs with a life cycle of 8 dpi. The expression of the anti-inflammatory factor IL-10 (**Figures 5I, J**) was significantly elevated during the near-death period. An imbalance between anti-inflammatory and proinflammatory factors results in a cytokine storm that induces acute inflammation. The expression of the innate immune factor IFN-α (**Figures 5K, L**) was significantly increased during the dying phase. The levels of the granulocyte chemokine IL-8 (**Figures 5M, N**) significantly rose at 6 dpi and declined to pre-attack levels at 8 dpi. The activating T and NK cytokine IL-12p40 (**Figures 5O, P**) did not show significant changes in domestic pigs with a life cycle of 8 days. In contrast, it was significantly elevated in pigs in the near-death period with a life cycle of 6 days. All these cytokines involved in innate immunity were suppressed, and the organism failed to produce adequate innate immune protection. The low level of IL-4 expression with undetectable IFN-γ levels (results not shown) suggested that the level of adaptive immunity in the organism was not high.

No abnormalities were found in pig No. 3 throughout the observation period. IL-1β (**Figure 5Q**) expression was significantly down-regulated after infection. IL-6 (**Figure 5R**) was significantly up-regulated at 4 dpi and then down-regulated, and there was no significant change in TNF-α (**Figure 5S**). IL-4 (**Figure 5T**) expression was also significantly down-regulated after infection, and IL-10 was not detected (results are for display). In terms of clinical signs and autopsy observations, there were no typical symptoms and tissue lesions were mild. In terms of inflammatory factors, there was no serious imbalance between pro-inflammatory and anti-inflammatory factors, and the inflammatory response was mild. It has been reported that significantly elevated levels of IL-10 in pigs infected with African swine fever predicted a lethal outcome (24, 25), whereas the remaining five pigs in this study showed a significant increase in levels during the near-death period. IFN-α (**Figure 5U**) was significantly down-regulated after infection and failed to cause a significant up-regulation in subsequent infections, a result that was opposite to that of the remaining five pigs that died. The expression of chemokine IL-8 (**Figure 5V**) and activator IL-12p40 (**Figure 5W**) was significantly up-regulated after infection, and the body was actively mobilizing immune cells to mature and activate, thus exerting an immune effect to antagonize the virus.

### 3.6 Transcriptional changes in PBMCs induced by ASFV infection of hosts

**Figure 6:**
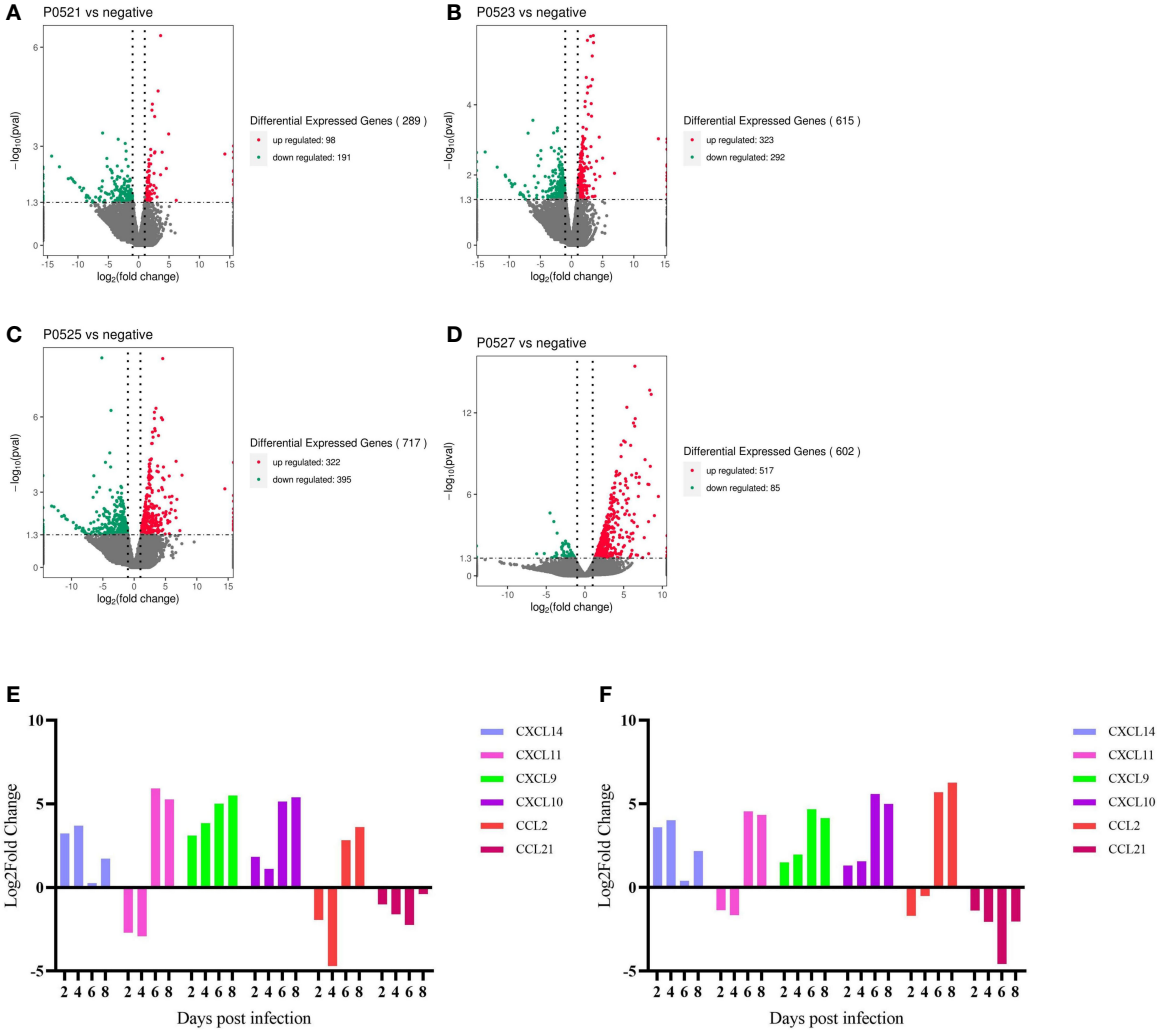
**(A-D)** Number of differential genes in PBMCs at different days of infection. **(E)** qpcr gene validation. **(F)** RNA-Seq gene changes.

Changes in genes at the transcriptional level were analyzed for different days of infection relative to those in the negative controls, with 289 differential genes at 2 dpi (**Figure 6A**), 615 differential genes at 4 dpi (**Figure 6B**), 717 differential genes at 6 dpi (**Figure 6C**), and 602 differential genes at 8 dpi (**Figure 6D**). We screened the cytokine genes measured by serum (absolute value of Log2FoldChange ≥ 1, pval < 0.05) for consistency in transcription and translation levels. Both IFN-α8 and IFN-α15 tended to be downregulated (no significant difference), whereas the serum results showed significant upregulation of IFN-α at 6 and 8 dpi. IFN-γ appeared to be upregulated (no significant difference), with very low or even undetectable amounts in the serum. IL-4 insignificantly changed but was significantly upregulated in the serum at 8 dpi. IL-6 was upregulated (no significant difference), with serum IL-8 upregulated at 6 dpi vs. 8 dpi (no significant difference) and significantly upregulated at 6 dpi but downregulated at 8 dpi in the serum (no significant difference). IL-10 transcriptional and translational levels were consistent and significantly upregulated at 6 and 8 dpi. IL12p40 was downregulated and then upregulated (no significant difference) but was significantly upregulated at 6 dpi in the serum. TNFAIP2 was significantly upregulated at 4 dpi versus 6 dpi, and TNFAIP3 was significantly upregulated at 4 dpi, Serum TNFα was significantly upregulated in the dying phase (**Supplementary File 1**).

The top 30 genes enriched to the most genes per term were analyzed, with the most genes enriched to biological process, with more genes enriched to immune system processes and protein targeting at 2 dpi (**Supplementary File 2A**), more genes enriched to positive–negative feedback regulation as well as other stimulus-feedback regulation at 4 dpi (**Supplementary File 2B**), and more genes enriched to stress response, external stimulus-response, immune response at 6 dpi, defense responses, and inter-tissue interactions (**Supplementary File 2C**) according to Gene Ontology (GO). The results at 8 dpi were similar to those at 6 dpi, except that more extracellular genes were enriched (**Supplementary File 2D**), and the remaining changes were similar to those at 6 dpi.

The top 20 most significant pathways enriched in each pathway were analyzed, and the enrichment of immune inflammation-related pathways was more significant at 2, 4, 6, and 8 dpi (**Supplementary Files 3A-D**). The immunoinflammation-related genes of the enriched pathways were screened (absolute value of Log2FoldChange ≥ 1, pval < 0.05). C1QA, C1QB, TCRα, and TCRβ on 2 dpi; C1QA, C1QB, TCRα, TCRβ, CD28, IL-1A, CCL4, TNFAIP3, and TRF9 on 4 dpi; C1QA, C1QB, C1QC, CD86, IL-10, CCL2, CCL4, CXCL9, CXCL10, CXCL11, CSF1, SLA-DOA, SLA-DMA, SLA-DMB, IgG, IRF7, and BCR on 6 dpi; C1QA, C1QB, C1QC, C4BP, C5aR, IL-1R2, IL-10, CCL2, CXCL10, CCL4, CCL3L1, CXCL11 XCL1, CXCR2, CCR10, CXCR1, CCR1, XCR1, TNFSF13, CSF1, IgA, IgG, IRF4, IRF7, CD14, and CD209 on 8 dpi were involved in two and above pathways (**Supplementary File 1**).

Comparing the changes in transcript levels of the six chemokines analyzed using qPCR with the results of RNA-seq (**Figures 6E, F**), CXCL14, CXCL9, and CXCL10 were upregulated from post infection to end of life, CXCL11 and CXCL2 were first downregulated at 2 and 4 dpi and then upregulated, and CCL21 was downregulated in all cases after infection.

### 3.7 Massive reduction in lymphocyte counts after ASFV infection

**Figure 7:**
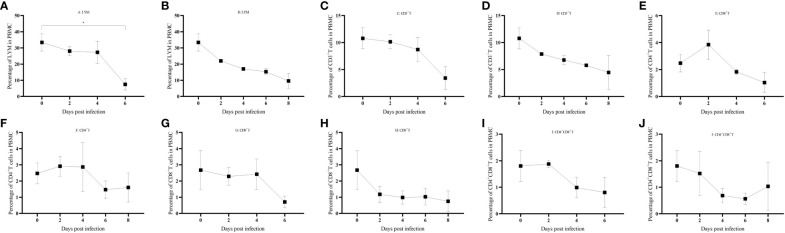
**(A–J)** Immune cell changes in different life processes. P < 0.05 (*), P < 0.01 (**), P < 0.001(***), and P < 0.0001 (****).

PBMCs were isolated from domestic pigs infected with HuB/HH/2019 ASFV (**Supplementary File 4**), and a clear trend of lymphocytes decreasing with days of infection was found by FSC-A and SSC-A set gates (**Figures 7A, B**). CD3+ T cells also gradually decreased in PBMCs (**Figures 7C, D**), and a transient rise in the latency period of CD4+ T cells was followed by the onset of a decline (**Figures 7E, F**), CD8+ T, CD4+CD8+ double-positive T cells all gradually decreased in PBMC (**Figures 7G, H**).

## 4 Discussion

African swine fever is a class I animal disease that needs to be reported to the World Organization for Animal Health (WOAH) once detected. Its widespread global epidemic has caused serious losses to the global pig farming industry, particularly since its introduction in China in 2018 (26). The virulent strain in this study was isolated in Hubei in 2019, and whole-genome sequencing revealed that the sequence was extremely similar to that of Georgia 2007/1 strain ASFV. HuB/HH/2019 strain ASFV-infected domestic pigs with typical symptoms of a strong genotype II strain (23).

Data on the patterns of virus carriage and detoxification in infected hosts can provide guidance for early diagnosis on farms. Our study found that infection with the Hub/2019 strain ASFV was detectable in oral saliva as early as 4 dpi, in blood at 4 dpi, and in feces at 6 dpi. Multiple tissues and lymph nodes were virulent after ASFV infection, with the highest viral loads in the spleen and lungs.

Only one pig secreted antibody while the rest of the pigs that died of acute morbidity did not secrete antibodies. From the RNA-seq results of this study (**Supplementary File 1**), antigen-presenting genes such as SLA were down-regulated in the PBMCs of the five pigs, and BCR was also down-regulated, resulting in a weakening of the ability of the B cells to recognize bound antigens and failing to differentiate into plasma cells to secrete antibodies. In addition, some antigen-presenting cells cause apoptosis (27) after infection with a strong virulent strain of the African swine fever virus, resulting in immune cells that cannot effectively recognize antigens to generate a humoral immune response. The final point is that B cells also cause apoptosis (28) after infection.

Cytokines, as a kind of protein secreted by a variety of cells, play important roles in immunity, inflammation, and tumor (29–31). And cytokine storm leads to an imbalance between pro-inflammatory and anti-inflammatory factors in the body, triggering an excessive inflammatory response, which can cause damage or even death to the body (32). The three criteria reported to measure cytokine storm are elevated circulating cytokine levels, acute systemic inflammation, and secondary organ dysfunction (usually kidney, liver, and lungs) caused by inflammation (33). Measured by these three criteria our study found that an imbalance between pro-inflammatory factors such as IL-1β, IL-6, and TNF-α and anti-inflammatory factors such as IL-4 and IL-10 triggers a systemic multiorgan inflammatory response, leading to fever, hemorrhagic swelling of various organs, and dysfunction in the body. Exogenous pyrogens (viruses, bacteria, parasites, steroids, antigen-antibody complexes, etc.) stimulate the organism and activate endogenous pyrogenic cells to release endogenous pyrogens, leading to an increase in body temperature (34). The rapid increase in body temperature after ASFV infection in domestic pigs may be caused by the increase in the release of endogenous pyrogens, such as IL-1β, IL-6, TNF-α, etc. (35, 36). Th1 and Th2 cells are a kind of helper T cells, the former is involved in cellular immunity and mainly secretes IFN- γ (37), while the latter is mainly involved in humoral immunity and secretes cytokines such as IL-4 (38). In this study, IFN- γ was not detected or was detected at very low levels throughout the pig’s life cycle, suggesting that NK cells and T cells that may secrete IFN-γ fail to activate effectively. IFN- γ plays an important role in defense against ASFV infection but is not the only indicator of the rate of protection generated. In immunization with certain deletion strains, IFN- γ levels were not elevated, but they were able to resist attack by their original strong strains (39–42). Certain weak strains infecting domestic pigs failed to protect against strong strains when IFN- γ levels were elevated (39, 42, 43). Although P30 antibody was not detected in the morbid pigs of this study, IL-4 levels were elevated significantly at 8 dpi in domestic pigs with a life cycle of 8 days, which is no longer able to promote antibody secretion in domestic pigs, and no significant increase in IL-4 levels was observed in domestic pigs with a life cycle of 6 days. In a study of ASFV and IL-10, a negative correlation was found between survival and IL-10 levels in domestic pigs (44). IL-10 levels remained stable in surviving pigs, while IL-10 levels increased significantly in dead pigs (24, 32, 42, 45–47). ASFV has evolved several strategies to inhibit IFN-α secretion, such as the pH240R protein that inhibits IFN-α production by disrupting STING oligomerization (48), pF778R that attenuates the I type interferon response (49). In this study, the IFN-α in the serum did not change in the early stage of infection, but the level of IFN-α increased significantly after the fever in domestic pigs. IFN-α is mainly involved in the innate immune response, and the inhibition of IFN-α secretion in the early stage of ASFV replication (50), which led to the avoidance of the innate immune response of ASFV, and then a large number of replications to infringe on the organism, and the level of IFN-α did not rise significantly until the end of life, but it was too late. IL-12 is a heterodimeric cytokine composed of two subunits, p35 and p40 (51, 52). It is mainly produced by antigen-presenting cells (APCs), especially monocytes and macrophages, in response to microbial pathogens (51, 52). There are fewer studies on the level of IL-12 changes in serum after ASFV infection. No changes in IL-12 levels were observed after ASFV-GΔ9GL/ΔUK, Netherland’86, HLJ/18-7GD, and HLJ/18 infections in domestic pigs (41, 44, 47). After SY18 infection, IL-12 levels increased rapidly (32). In this study, no significant changes in IL-12 were seen in domestic pigs with a life cycle of 8 days, whereas domestic pigs with a life cycle of 6 days showed a significant increase at 6 dpi.IL-8 is also known as CXCL8. CXCL8 is widely regarded as a potent neutrophil chemotactic agent that promotes the aggregation of neutrophils and other granulocytes to the site of infection (53). In addition, this chemokine triggers neutrophil degranulation and enhances their phagocytosis (51, 54). IL-8, similarly to IL-12, is found in varying serum levels in hosts infected with different strains. Benin97/1, OURT88/3, Pret4Δ9GL, ASFV-G-Δ9GL/ΔUK, Netherland ‘86 and other strains did not show significant changes in IL-8 levels after infection of the host (40, 41, 44, 55). In contrast, strains such as Armenia07, Armenia08, and SY18 showed elevated IL-8 levels after host infection (32, 56, 57). In the present study, domestic pigs with a life cycle of 6 and 8 days had the highest levels at 6 dpi. However, domestic pigs with a life cycle of 8 days had rapidly decreasing levels at 8 dpi.

Infection with the HuB/HH/2019 strain of ASFV results in an imbalance between pro- and anti-inflammatory factors, leading to a cytokine storm manifested as fever, multiple tissue swelling and hemorrhage, and organ dysfunction. Factors involved in the innate immune response were suppressed and failed to protect the pig. IL-4 was elevated in the dying phase, and IFN-γ was not detected, suggesting that humoral and cellular immunity were not effectively activated. The lack of detection of the p30 antibody and the massive reduction in lymphocytes in PBMCs seems to provide more evidence of the suppression of adaptive immunity. The combination of acute inflammation and immunosuppression results in the inability of the organism to resist ASFV, leading to a lethal outcome.

Transcriptome RNA-seq is a recently developed technology that can elucidate the pathogenic and immune mechanisms of pathogens by revealing dynamic changes in the genomes of pathogens and systematic changes in host genes during pathogen infection (58). The complement level was significantly increased at 2, 4, 6, and 8 dpi. The complement system is an important component of innate immunity, which plays an important role in antimicrobial activity and inflammation, and C1 participates in the classical activation pathway (59). The chemokine family showed a significant increase after infection. Chemokines are important mediators of acute inflammation and components in generating primary and secondary adaptive cellular and humoral immune responses. Chemokine receptors (CCR1, CCR10, CXCR1, CXCR10, and XCR1) were significantly upregulated in this study, and these receptors are expressed on the cell surface of granulocytes, T cells, plasma cells, monocytes, and macrophages, and perform functions such as lymphangiogenesis, immune cell transport, and innate and adaptive immunity (60). Chemokines (CCL2, CCL3L1, CCL4, CXCL9, CXCL10, CXCL11, and XCL1) were also significantly upregulated after infection. These chemokines mainly play a role in the migration of monocytes, Th1 cells, NK cells, macrophages, and other cells (60). Therefore, we hypothesized that genes such as complement, interleukins, chemokines, interferon modulators, T-cell receptors, B-cell receptors, SLA-like molecules, CD molecules, and immunoglobulins play important roles in resisting viral infection after HuB/HH/2019 ASFV infection in domestic pigs.

African swine fever has evolved multiple immune escape mechanisms to ensure its replication. When the viral infection is in the latent phase, innate immunity fails to effectively detect the invasion of the virus, providing a safe and effective environment for virus replication (61). When the virus is released in large quantities, on the one hand, the increase of apoptotic factors leads to a large reduction of lymphocytes (62), and on the other hand, the cells involved in antigen presentation (B-cells, DC-cells, etc.) are reduced so that lymphocytes do not recognize the antigen effectively, and the immune system is not able to defend against the virus, which ultimately leads to lethal results (27). African swine fever mainly infects the monocyte/macrophage system (63) and does not infect lymphocytes, but lymphocytes in infected pigs die in large numbers (28), suggesting that lymphocyte apoptosis is indirectly rather than directly affected by African swine fever. TNF-α has an apoptotic effect, and some articles report that the apoptosis of lymphocytes is affected by TNF-α (62). Therefore, the dramatic increase in inflammatory and pro-apoptotic factors secreted by macrophages infected with African swine fever virus is an important cause of the cytokine storm and lymphocytopenia.

## Data availability statement

The datasets presented in this study can be found in online repositories. The names of the repository/repositories and accession number(s) can be found below: PRJNA1054340 (SRA).

## Ethics statement

The animal studies were approved by Animal Welfare Ethics Committee of the China Institute of Veterinary Drug Control, China Institute of Veterinary Drug Control. The studies were conducted in accordance with the local legislation and institutional requirements. Written informed consent was obtained from the owners for the participation of their animals in this study.

## Author contributions

XZZ: Writing – original draft, Writing – review & editing. GP: Writing – original draft, Writing – review & editing. JZ: Writing – original draft, Writing – review & editing. QZ: Writing – original draft, Writing – review & editing. YZ: Writing – original draft, Writing – review & editing. YX: Writing – original draft, Writing – review & editing. LX: Writing – original draft, Writing – review & editing. FL: Writing – original draft, Writing – review & editing. YJX: Writing – original draft, Writing – review & editing. YL: Writing – original draft, Writing – review & editing. CW: Writing – original draft, Writing – review & editing. ZW: Writing – original draft. HW: Writing – original draft, Writing – review & editing. XQZ: Writing – original draft, Writing – review & editing.
