# Supplementary material for: Infection of domestic pigs with a genotype II potent strain of ASFV causes cytokine storm and lymphocyte mass reduction
Source: Front Immunol. 2024 Apr 18;15:1361531. doi: 10.3389/fimmu.2024.1361531 (PMC11064794; doi:10.3389/fimmu.2024.1361531)
Supplement: Supplementary file 1 [file DataSheet_1.docx]

A
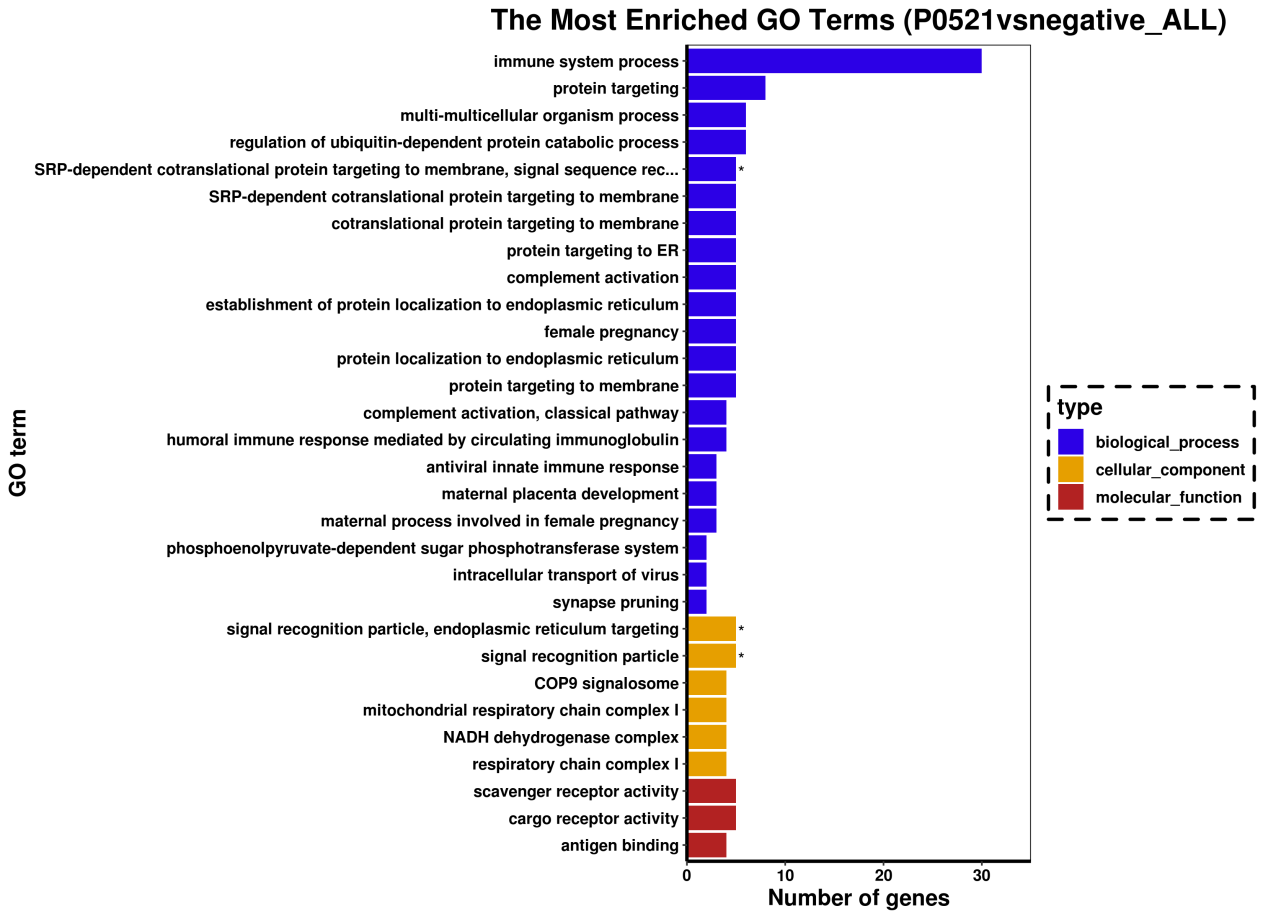


B


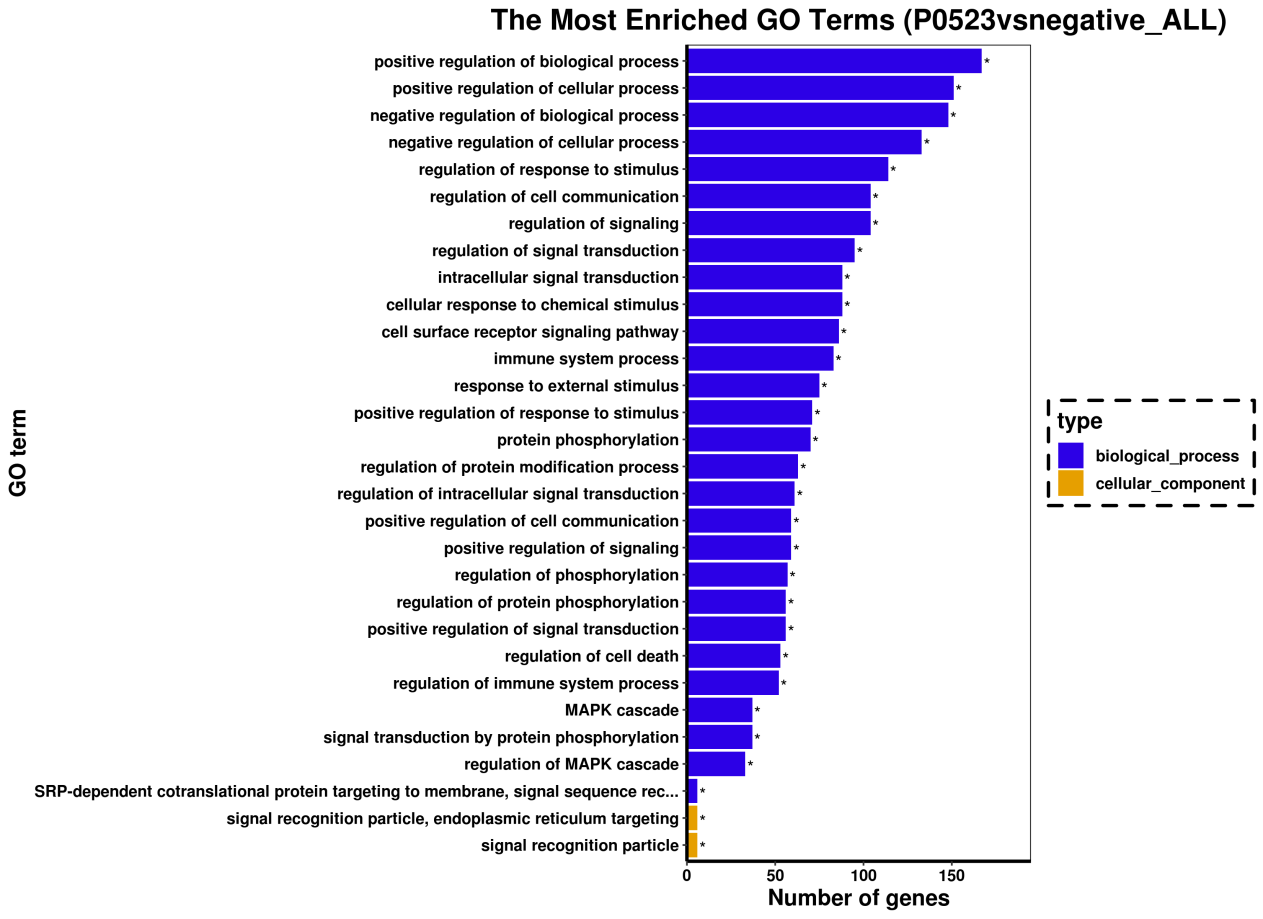


C
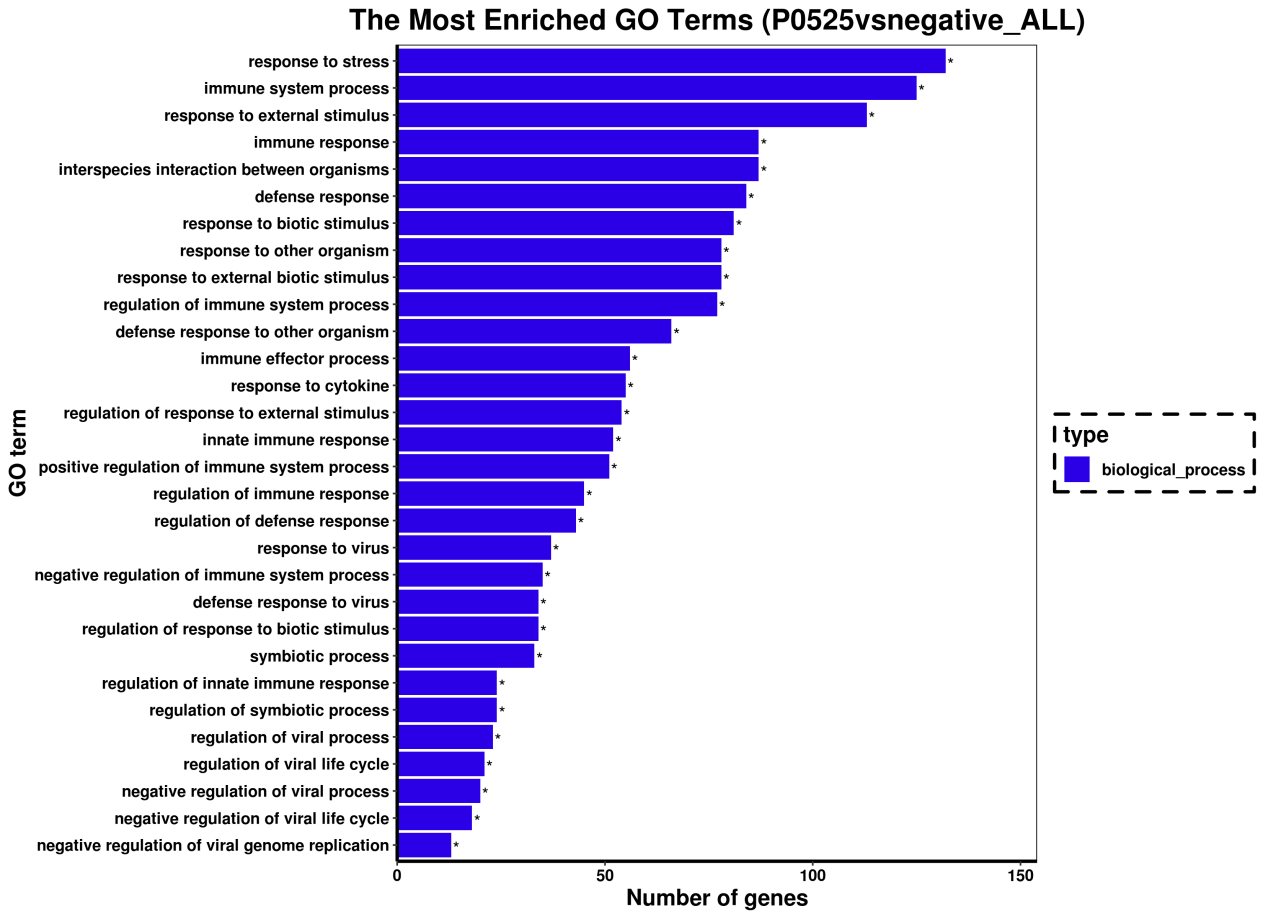


D
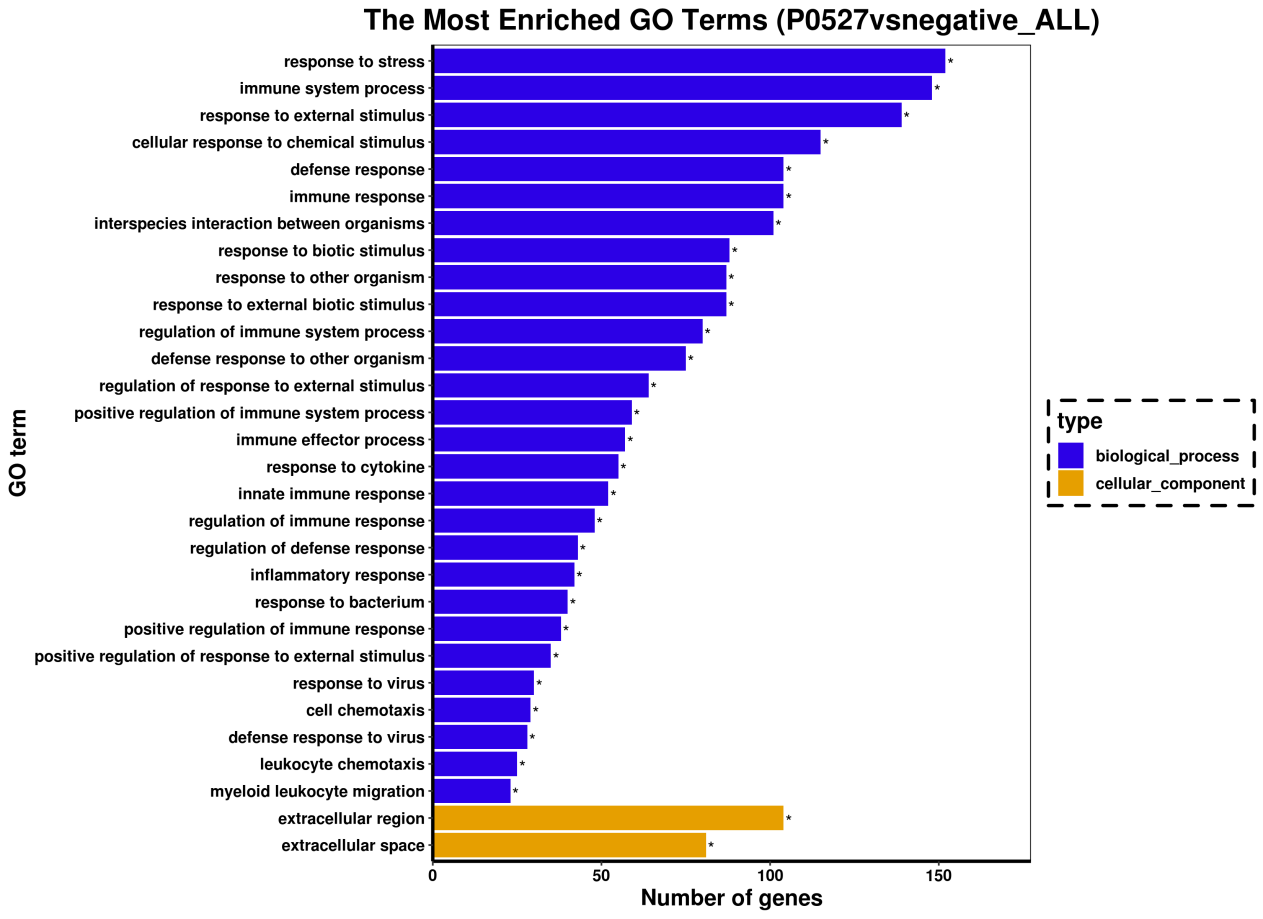


KEGG enrichment analyses for 2, 4, 6, and 8 dpi (**A**, **B**, **C**, and **D**, respectively). Rich factor refers to the ratio of the number of differentially expressed genes enriched in the pathway to the number of annotated genes. The larger the value of the rich factor, the greater the degree of enrichment. The Q-value (range of 0–1) is the p-value corrected by multiple hypothesis testing. The closer the q-value is to zero, the more significant the enrichment is.
