# Supplementary material for: Infection of domestic pigs with a genotype II potent strain of ASFV causes cytokine storm and lymphocyte mass reduction
Source: Front Immunol. 2024 Apr 18;15:1361531. doi: 10.3389/fimmu.2024.1361531 (PMC11064794; doi:10.3389/fimmu.2024.1361531)
Supplement: Supplementary file 2 [file DataSheet_2.docx]

A


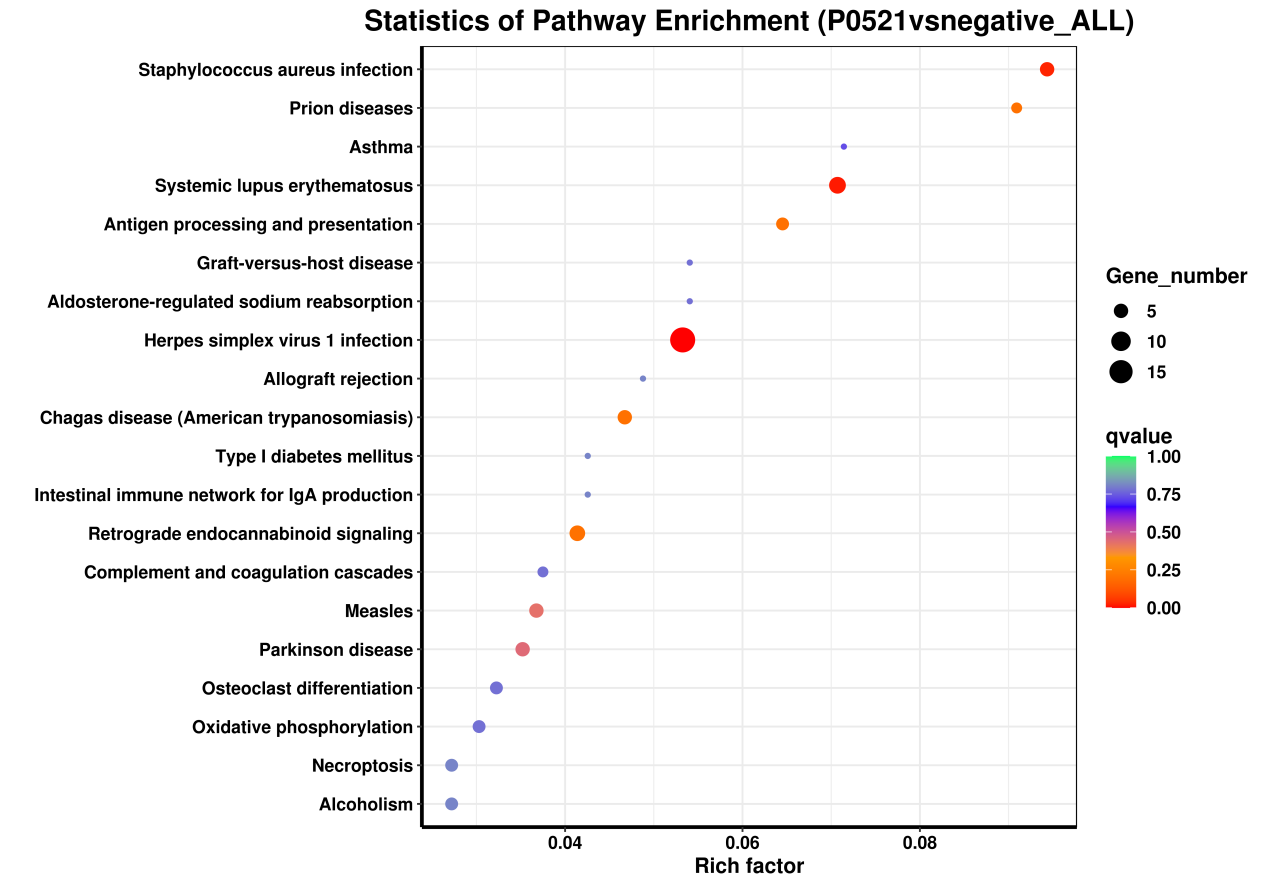


B


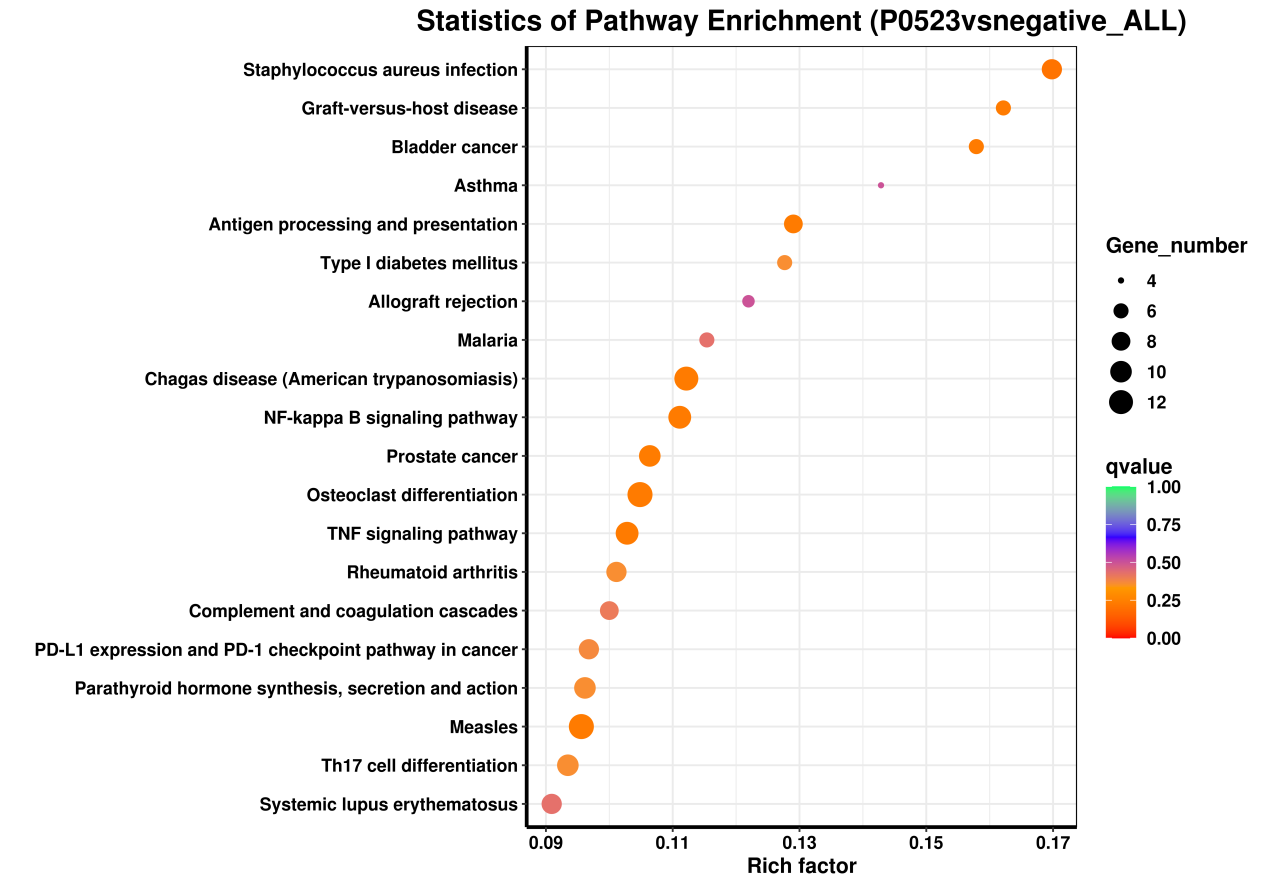


C


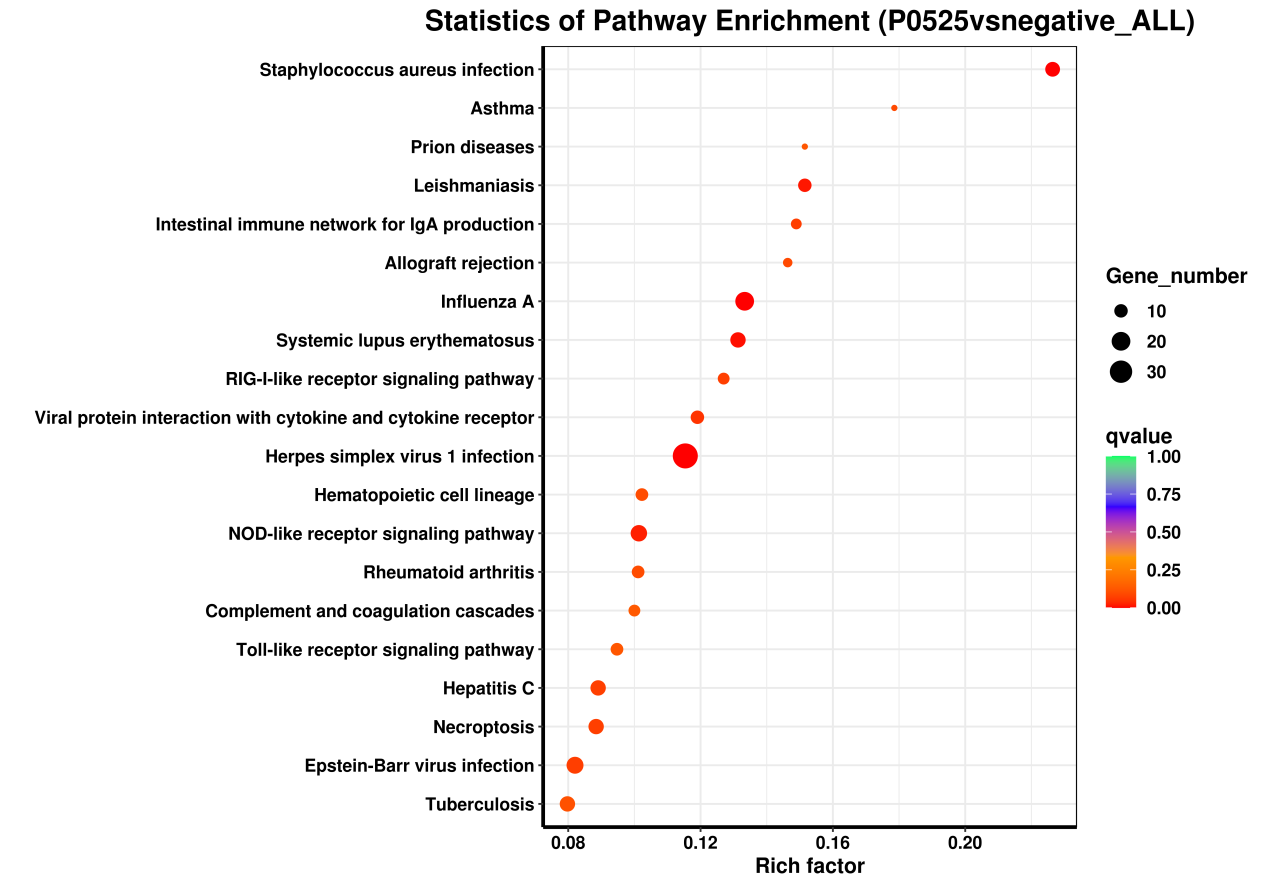


D


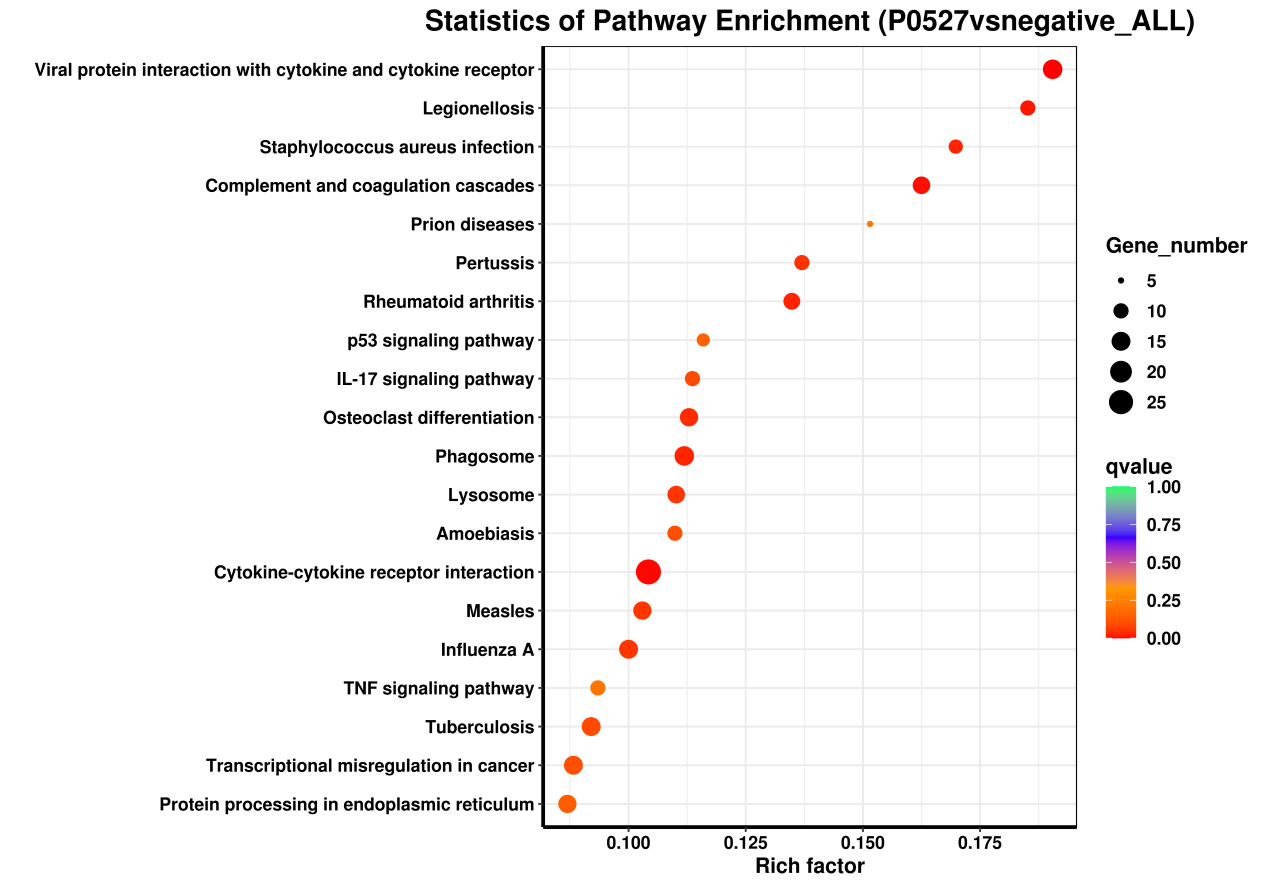


GO enrichment plots for 2, 4, 6, and 8 dpi (**A**, **B**, **C**, and **D**, respectively). Blue represents the biological process, yellow represents cellular composition, and red represents molecular function. The horizontal and vertical tables represent the number of enriched genes. The vertical coordinates represent the function of the GO term.
