# Supplementary figures and images for: Infection of domestic pigs with a genotype II potent strain of ASFV causes cytokine storm and lymphocyte mass reduction
Source: Front Immunol. 2024 Apr 18;15:1361531. doi: 10.3389/fimmu.2024.1361531 (PMC11064794; doi:10.3389/fimmu.2024.1361531)

Control1
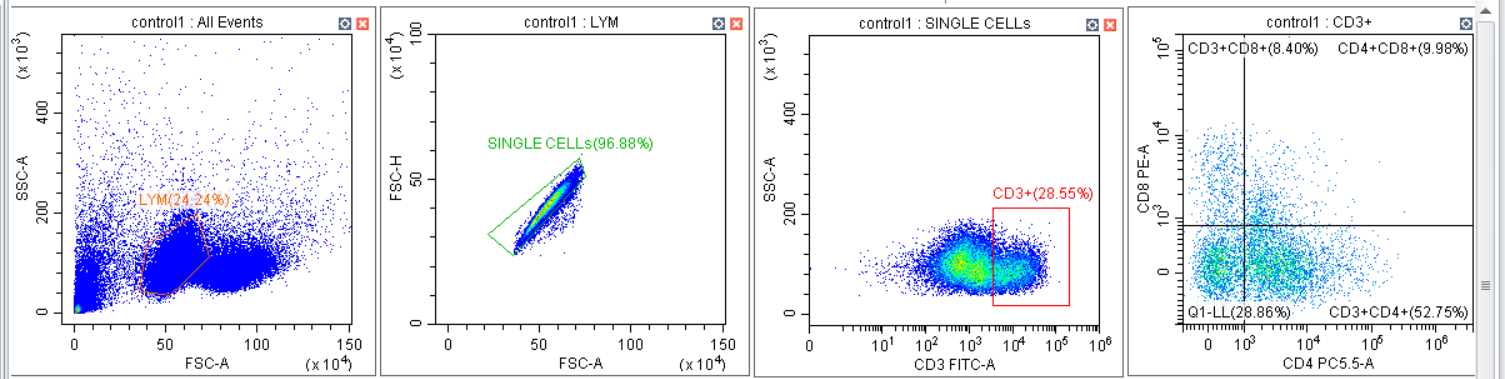


Control2


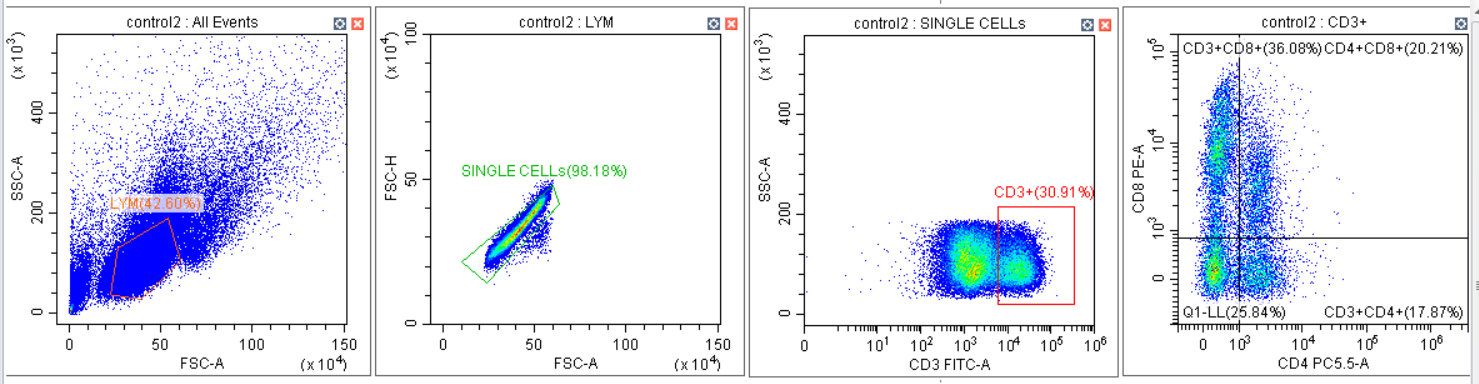


Control3


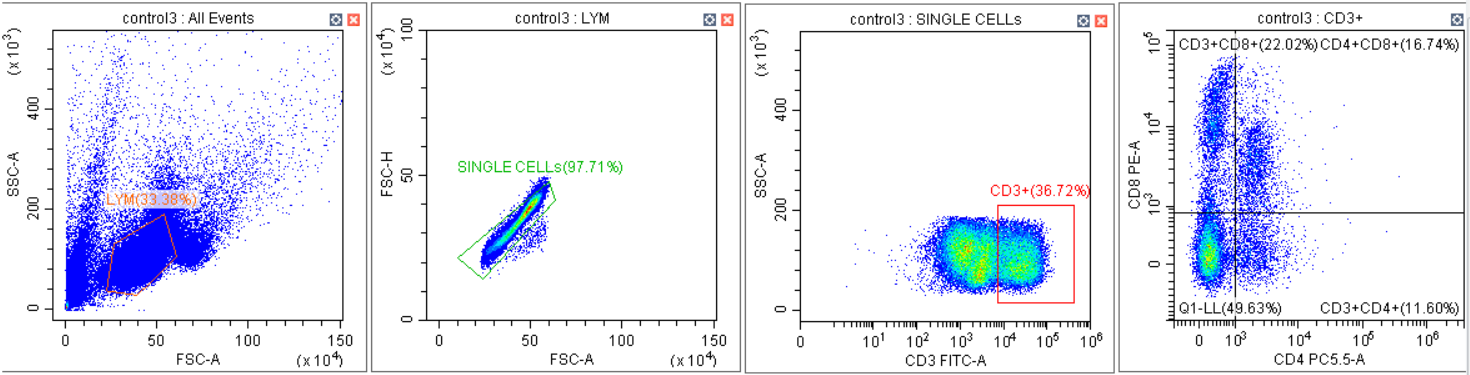


2# 2dpi


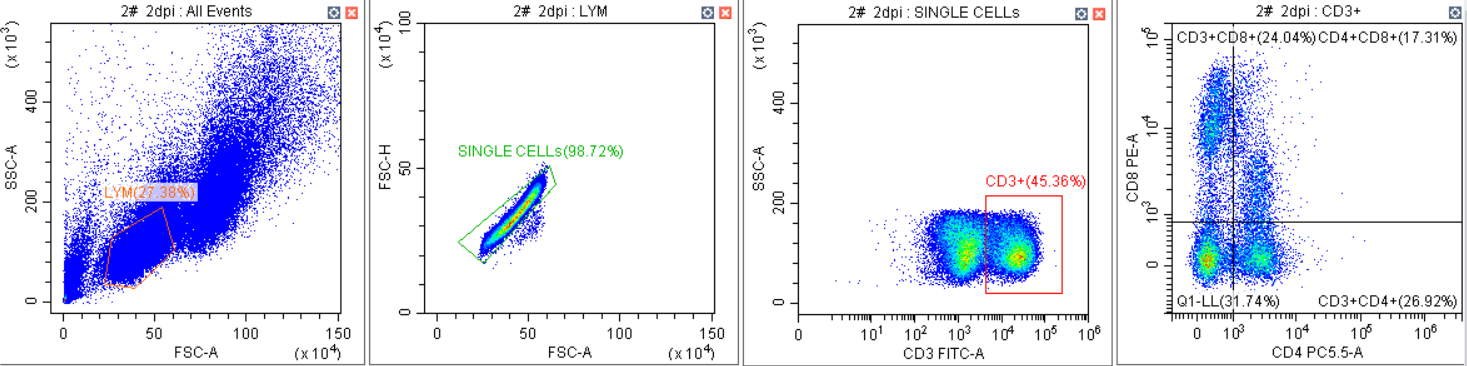


5# 2dpi


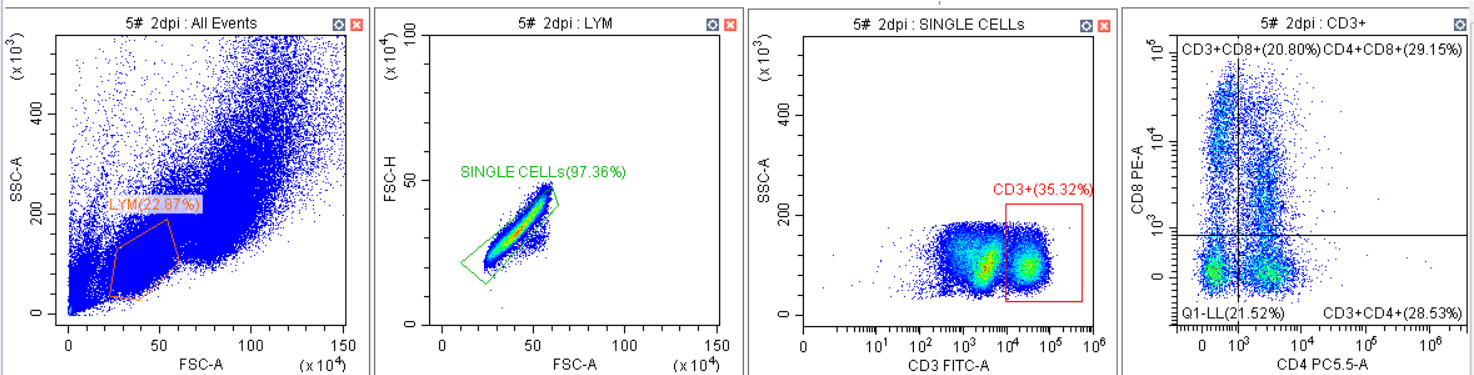


7# 2dpi


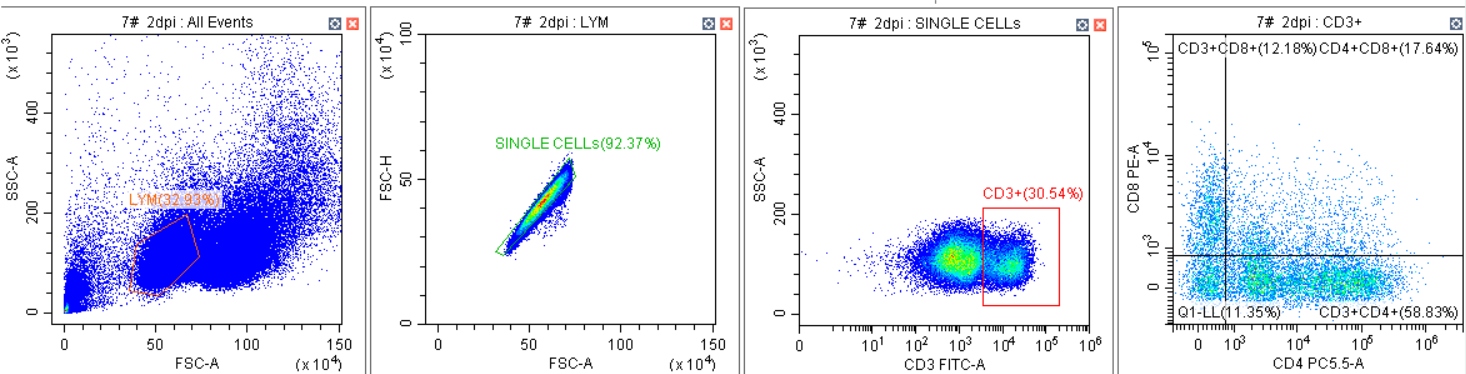


8# 2dpi


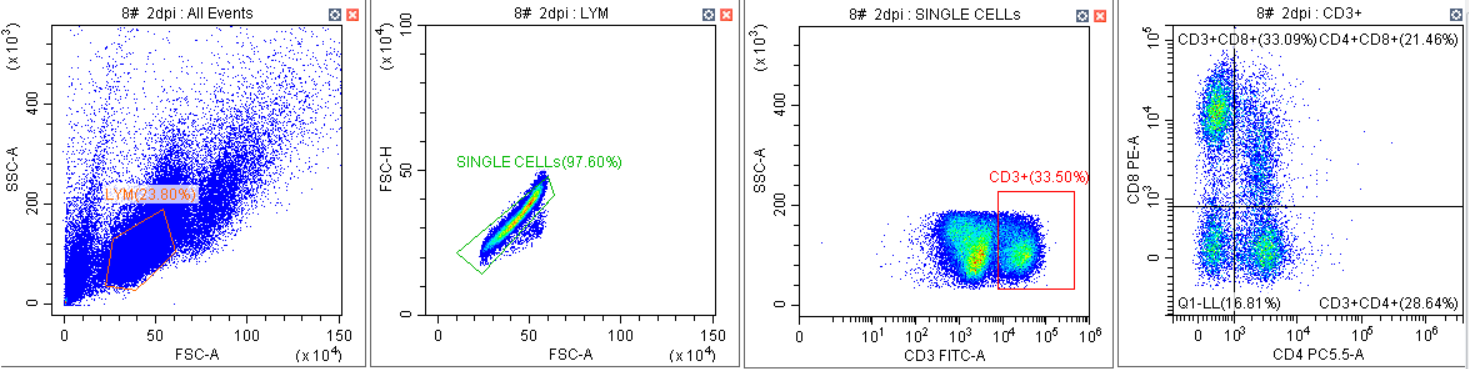


9# 2dpi


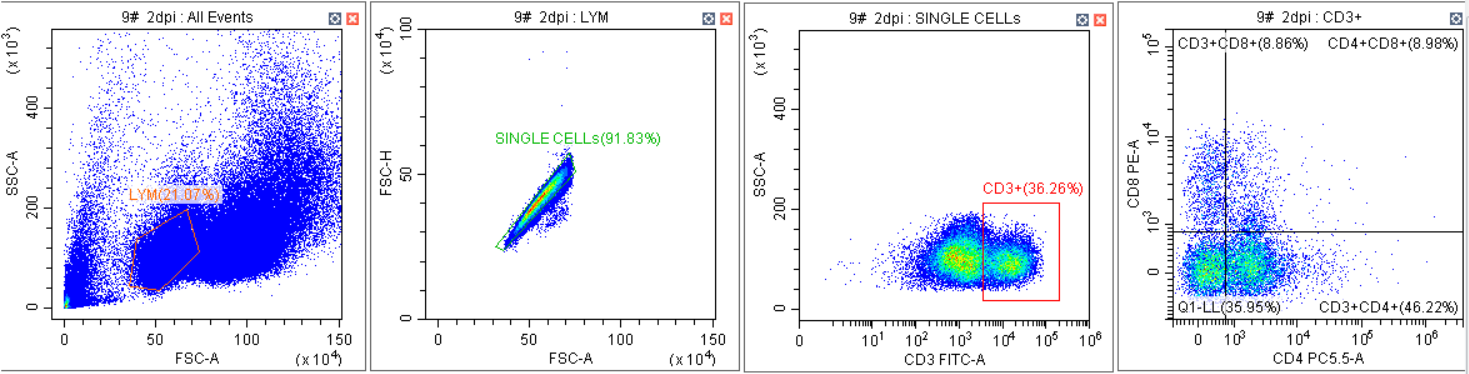


2# 4dpi


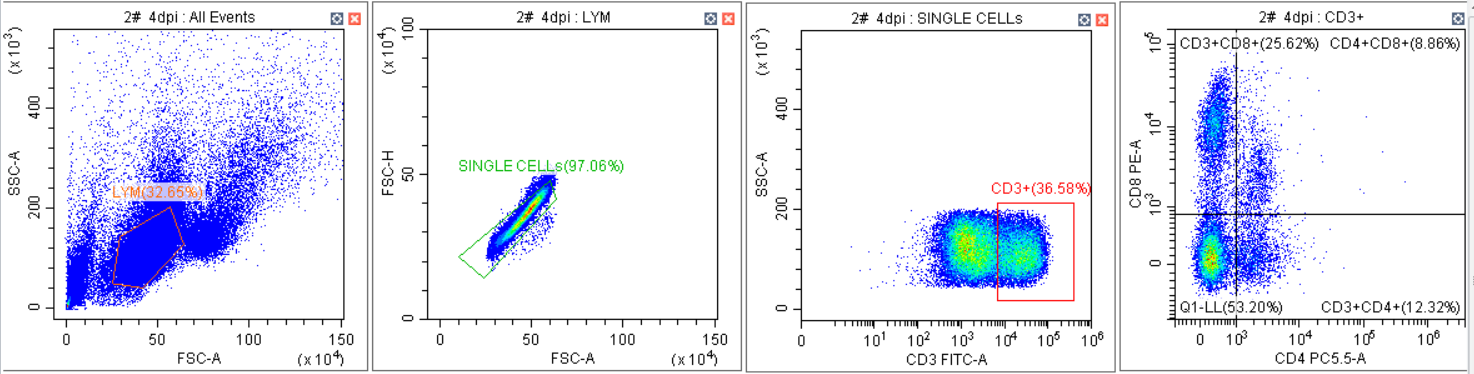


5# 4dpi


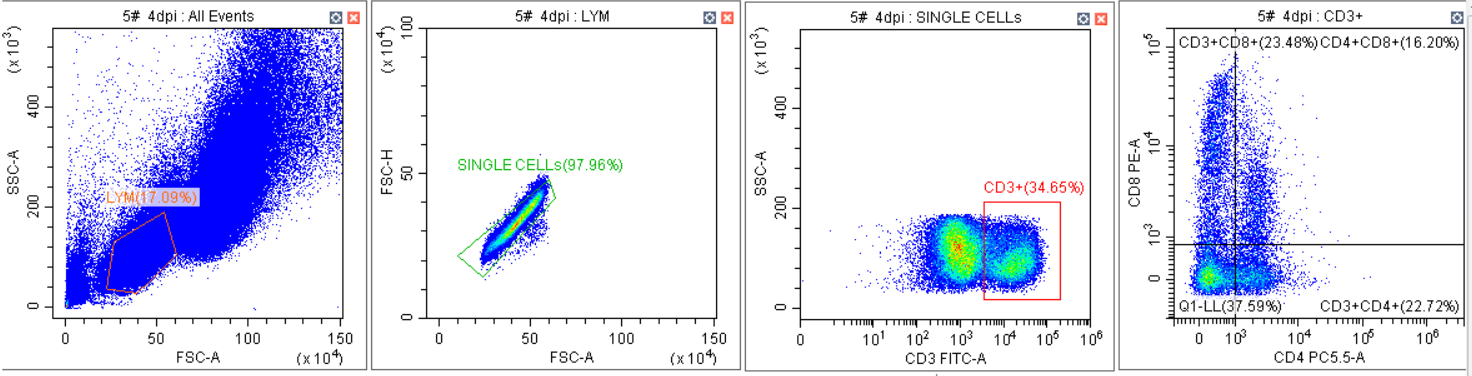


7# 4dpi


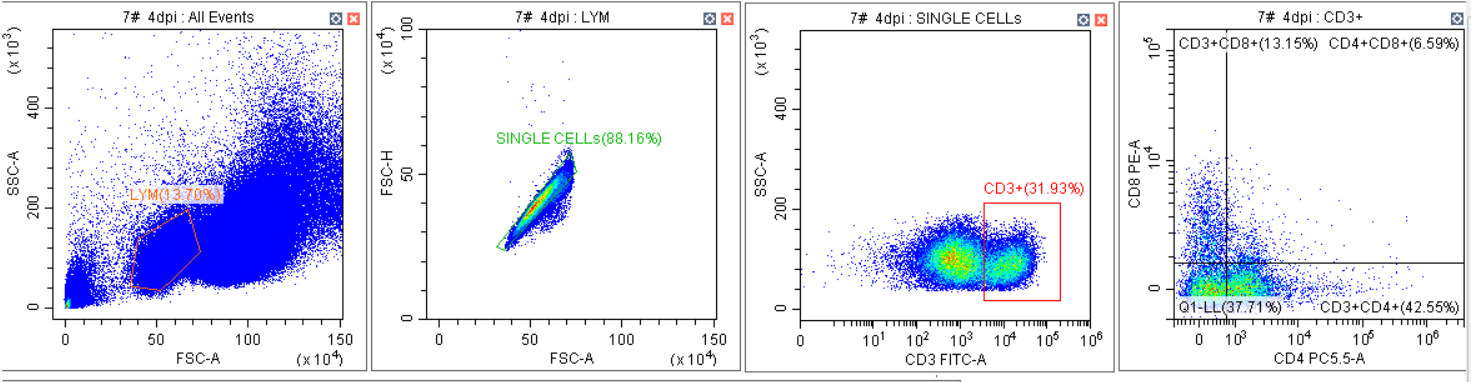


8# 4dpi


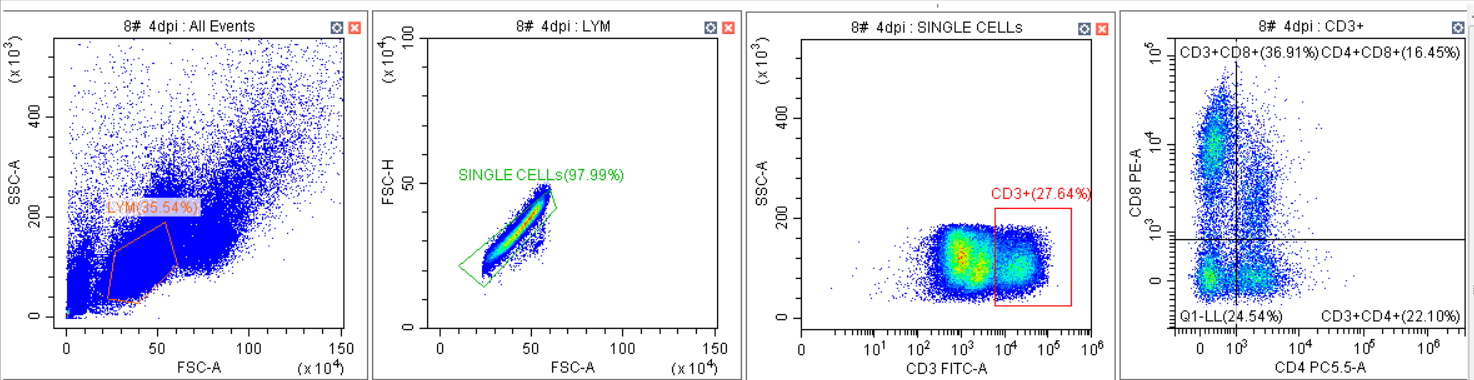


9# 4dpi


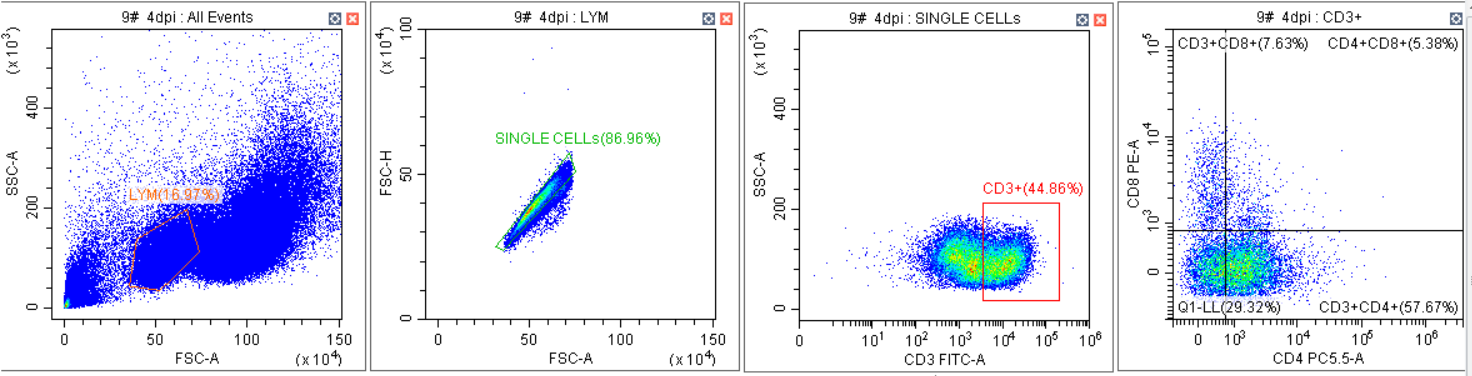


2# 6dpi


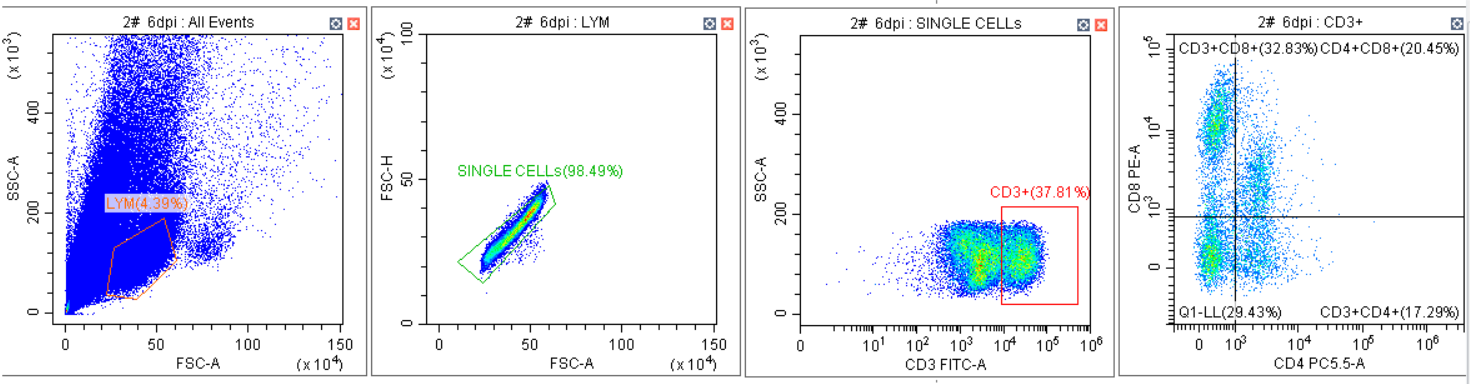


5# 6dpi


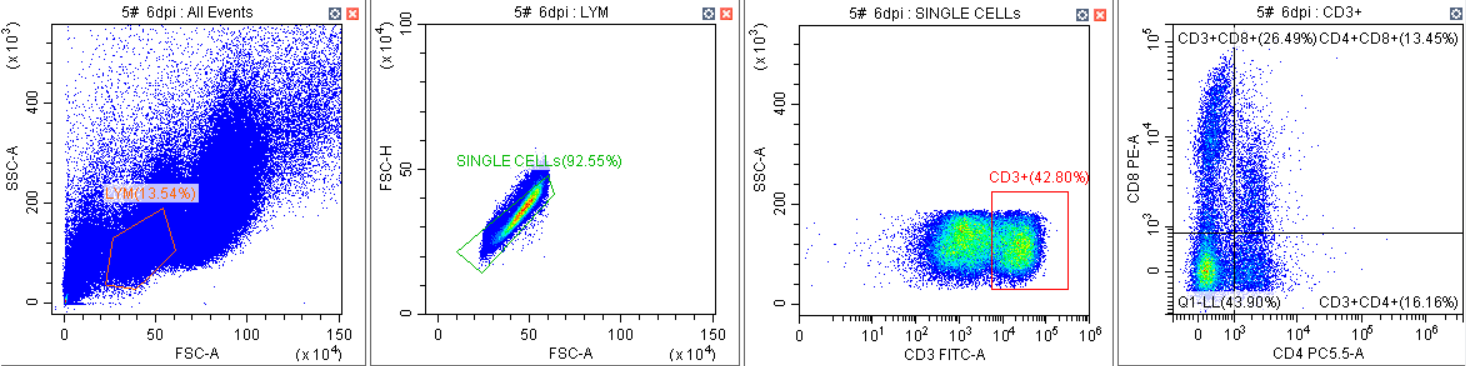


7# 6dpi


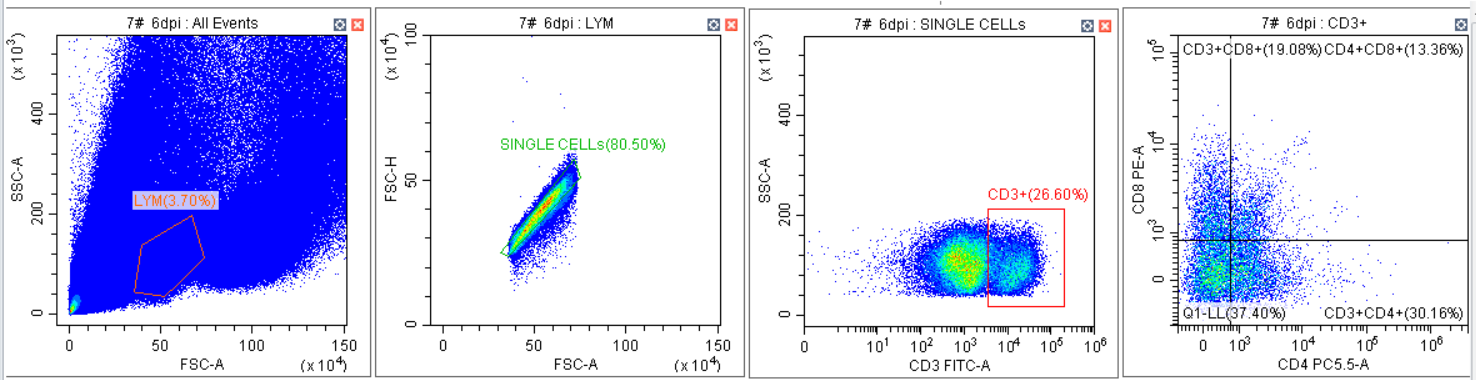


8# 6dpi


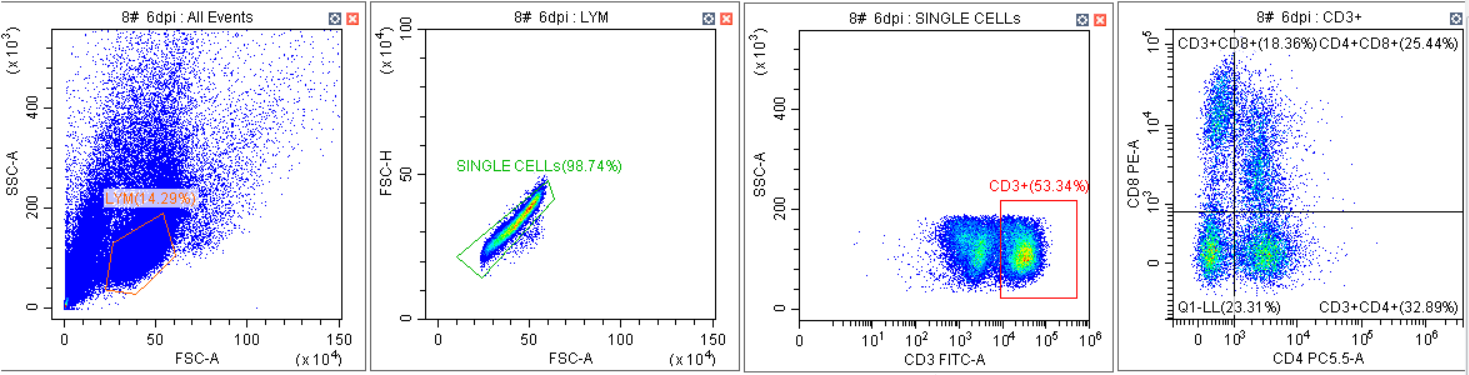


9# 6dpi


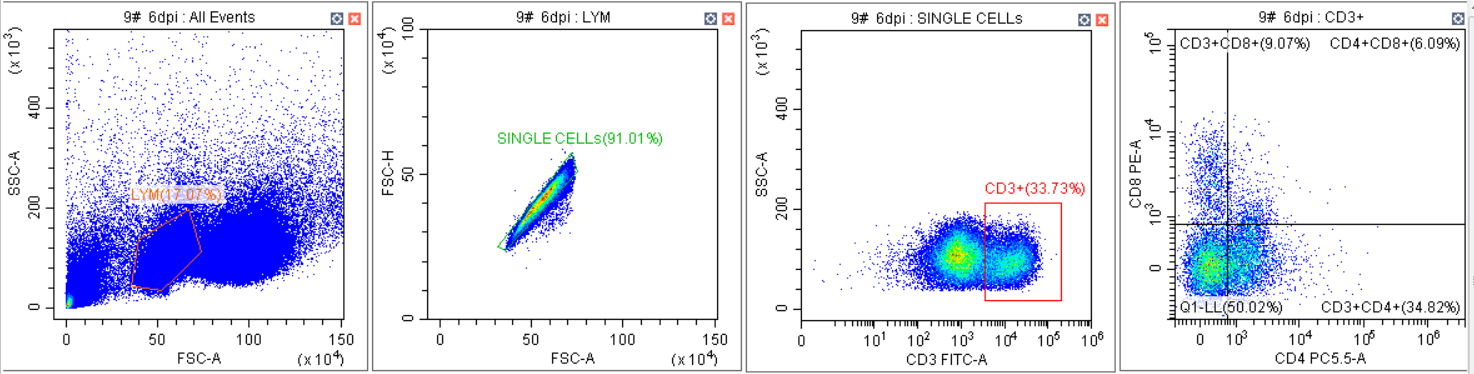


5# 8dpi


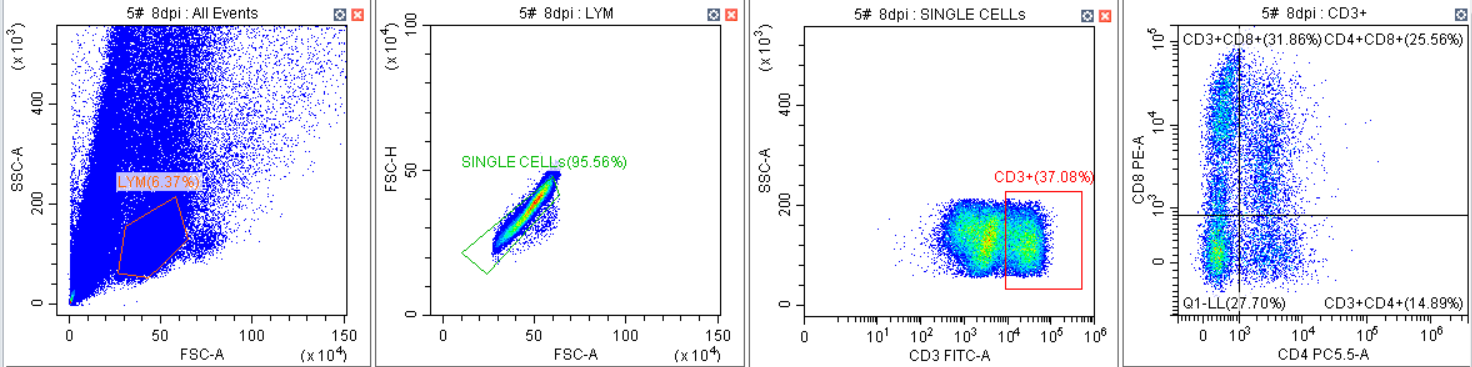


9# 8dpi


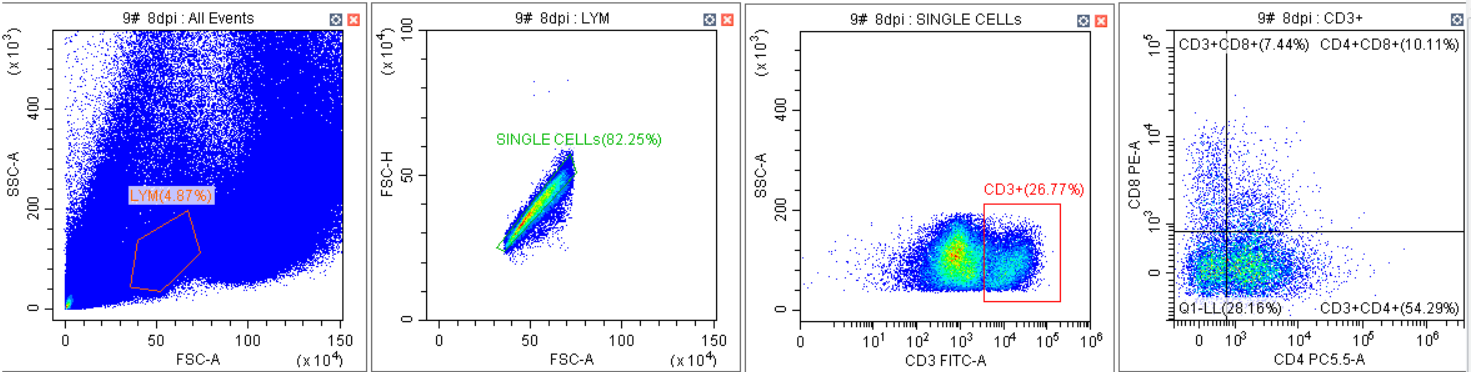

Supplement: Supplementary file 3 [file DataSheet_3.docx]
